# An Infection-Tolerant Mammalian Reservoir for Several Zoonotic Agents Broadly Counters the Inflammatory Effects of Endotoxin

**DOI:** 10.1128/mBio.00588-21

**Published:** 2021-04-13

**Authors:** Gabriela Balderrama-Gutierrez, Ana Milovic, Vanessa J. Cook, M. Nurul Islam, Youwen Zhang, Hippokratis Kiaris, John T. Belisle, Ali Mortazavi, Alan G. Barbour

**Affiliations:** a Department of Developmental and Cell Biology, School of Biological Sciences, University of California Irvine, Irvine, California, USA; b Department of Microbiology & Molecular Genetics, School of Medicine, University of California Irvine, Irvine, California, USA; c Department of Microbiology, Immunology, & Pathology, College of Veterinary Medicine & Biomedical Sciences, Colorado State University, Fort Collins, Colorado, USA; d Department of Drug Discovery & Biomedical Sciences, College of Pharmacy, University of South Carolina, Columbia, South Carolina, USA; e Peromyscus Genetic Stock Center, University of South Carolina, Columbia, South Carolina, USA; f Department of Medicine, School of Medicine, University of California Irvine, Irvine, California, USA; g Department of Ecology & Evolutionary Biology, School of Biological Sciences, University of California Irvine, Irvine, California, USA; University of Washington

**Keywords:** *Borrelia*, Lyme disease, *Mus musculus*, *Peromyscus leucopus*, RNA-seq, innate immunity, lipopolysaccharide, metabolomics

## Abstract

Animals that are competent reservoirs of zoonotic pathogens commonly suffer little morbidity from the infections. To investigate mechanisms of this tolerance of infection, we used single-dose lipopolysaccharide (LPS) as an experimental model of inflammation and compared the responses of two rodents: Peromyscus leucopus, the white-footed deermouse and reservoir for the agents of Lyme disease and other zoonoses, and the house mouse Mus musculus. Four hours after injection with LPS or saline, blood, spleen, and liver samples were collected and subjected to transcriptome sequencing (RNA-seq), metabolomics, and specific reverse transcriptase quantitative PCR (RT-qPCR). Differential expression analysis was at the gene, pathway, and network levels. LPS-treated deermice showed signs of sickness similar to those of exposed mice and had similar increases in corticosterone levels and expression of interleukin 6 (IL-6), tumor necrosis factor, IL-1β, and C-reactive protein. By network analysis, the M. musculus response to LPS was characterized as cytokine associated, while the *P. leucopus* response was dominated by neutrophil activity terms. In addition, dichotomies in the expression levels of arginase 1 and nitric oxide synthase 2 and of IL-10 and IL-12 were consistent with type M1 macrophage responses in mice and type M2 responses in deermice. Analysis of metabolites in plasma and RNA in organs revealed species differences in tryptophan metabolism. Two genes in particular signified the different phenotypes of deermice and mice: the Slpi and Ibsp genes. Key RNA-seq findings for *P. leucopus* were replicated in older animals, in a systemic bacterial infection, and with cultivated fibroblasts. The findings indicate that *P. leucopus* possesses several adaptive traits to moderate inflammation in its balancing of infection resistance and tolerance.

## Abstract (precis)

Animals that are natural carriers of pathogens that cause human diseases commonly manifest little or no sickness as a consequence of infection. Examples include the deer mouse, Peromyscus leucopus, which is a reservoir for Lyme disease and several other disease agents in North America, and some types of bats, which are carriers of viruses with pathogenicity for humans.

## INTRODUCTION

Peromyscus leucopus, the white-footed deermouse, is a major reservoir for several zoonotic agents (reviewed in reference 1). The infections include Lyme disease, as well as varieties of anaplasmosis, babesiosis, relapsing fever, ehrlichiosis, and viral encephalitis (2). P. leucopus is broadly distributed across the eastern and central United States (3, 4), adapted to a variety of habitats, and an important host for the tick vectors of disease (5). Its immune system and other defenses keep the pathogens at bay, but infections persist (6, 7), thereby increasing the likelihood that a tick acquires the microbe during its blood meal (8). Where enzootic transmission is pervasive, the majority of *P. leucopus* animals live parasitized by one or more of these pathogens (9–11). If there is a fitness cost, scrutiny has not revealed it in the field (11) or laboratory (12–14).

The term for persistent infection with minimal morbidity is infection tolerance (15–17) and distinguished from the immune system’s “tolerance” of self-antigens (18). In both contexts, “tolerance” conveys a moderation of the host response and avoidance of injury. Infection tolerance has been observed mainly at the organismal level, for instance, by signs of illness, disability, and fitness measures. These presumably are explained by events at the cellular and molecular levels (19, 20), but these have not been fully explored.

*P. leucopus*’ tolerance of infection is matched by that of another deermouse, Peromyscus maniculatus, a reservoir for a hantavirus (21, 22). *P. maniculatus* has also been experimentally infected with the coronavirus (CoV) disease 2019 (COVID-19) virus and observed to transmit it to cagemates. Yet infected animals displayed only moderate pathology and recovered within a few days (23, 24). Other examples of the tolerance phenomenon are found among bat species implicated as reservoirs for severe acute respiratory syndrome (SARS)-CoV, Ebola virus, Nipah virus, and Hendra virus (25, 26). These deermice and bats exhibit a trade-off between defensive processes that check pathogen proliferation and processes that limit collateral damage from those defenses. The result is a state of persistent infection with limited disability.

The innate and adaptive host defenses that in concert resist and neutralize pathogens are well known. Less understood are mechanisms on the other side of the trade-off (27), namely, those that curb sickness and illness due to maladaptive inflammation (28). *P. leucopus* is well suited for such investigations of infection tolerance (29–31). Colloquially called “mice,” the genus *Peromyscus*, along with hamsters and voles, belong to the family Cricetidae and not the family Muridae, which includes the house mouse, Mus musculus (32). We have previously sequenced the genome of *P. leucopus*, annotated its transcriptome using transcriptome sequencing (RNA-seq), and characterized its gastrointestinal microbiota (33–35). There was little difference by RNA-seq between the blood of *P. leucopus* experimentally infected with the Lyme disease agent Borreliella burgdorferi and that of uninfected controls (34), a finding consistent with the mildness of deermouse infections with this pathogen (36). Accordingly, we looked to alternatives that would more robustly elicit inflammation.

To that end, we compared the response to a single dose of bacterial lipopolysaccharide (LPS) of P. leucopus to that of M. musculus. This endotoxin leads to inflammation through its binding to a pattern recognition receptor and complex ensuing cascades (37). In its higher doses, injected LPS elicits a sepsis-like state that shares manifestations, such as fever and shock, with a variety of serious infections, including those caused by some protozoa and viruses, as well as bacteria. Tolerance of LPS is a long-recognized phenomenon (38), but this third usage of “tolerance” refers to a diminution in the severity of the response through cumulative previous exposures of animals or isolated cells to LPS (39, 40), rather than an inherent disposition of a naive animal to survive a toxic dose. Some spontaneous and engineered Mendelian traits do render a mouse less susceptible to LPS through an alteration of Toll-like receptor 4 (TLR4) for LPS or in key downstream mediators, such as MyD88, in the cascade (41, 42). In these examples, this at the cost of a compromised innate defense against bacterial infection. For this and other reasons, we thought it unlikely that a single gene or locus accounted for the more nuanced interplays between complex contending forces in *Peromyscus* and other reservoirs for a variety of disease agents.

The simple experimental system of *in vivo* LPS exposure is a proxy for acute systemic infection but without the confounding variables of changing numbers of the microbe and the inevitable appearance of acquired immunity. Our working assumption is that early events in the response are determinants of the eventual outcomes of the infection, whether assessed from the perspective of pathogen burden or host disability. In the course of the study, we identified several pathways involved with inflammation, oxidative stress, phagocytosis, and metabolism that distinguished *P. leucopus* and *M. musculus* in their responses to LPS. These have implications for studies of other vertebrate reservoirs of the agents of emerging zoonoses.

## RESULTS

### Susceptibility of *P. leucopus* to LPS.

We found no published reports on the systemic effects of different doses of LPS in any *Peromyscus* species, but there were several on the susceptibility of *M. musculus* to LPS (43–50). The 50% lethal dose (LD_50_) for *M. musculus* was in the range of 5 to 20 mg of LPS per kg of body weight, with an average across inbred and outbred mice of ∼15 mg/kg. Death usually occurred between 12 and 48 h after injection. We previously found that adult BALB/c mice injected intraperitoneally (i.p.) with 10 mg LPS per kg body weight manifested reduced activity and ruffled fur at 4 h, with concomitant elevations in serum concentrations of tumor necrosis factor alpha (TNF-α), interleukin 6 (IL-6), and IL-10 by immunoassay, but all animals survived up to 12 h (51).

**FIG 1:**
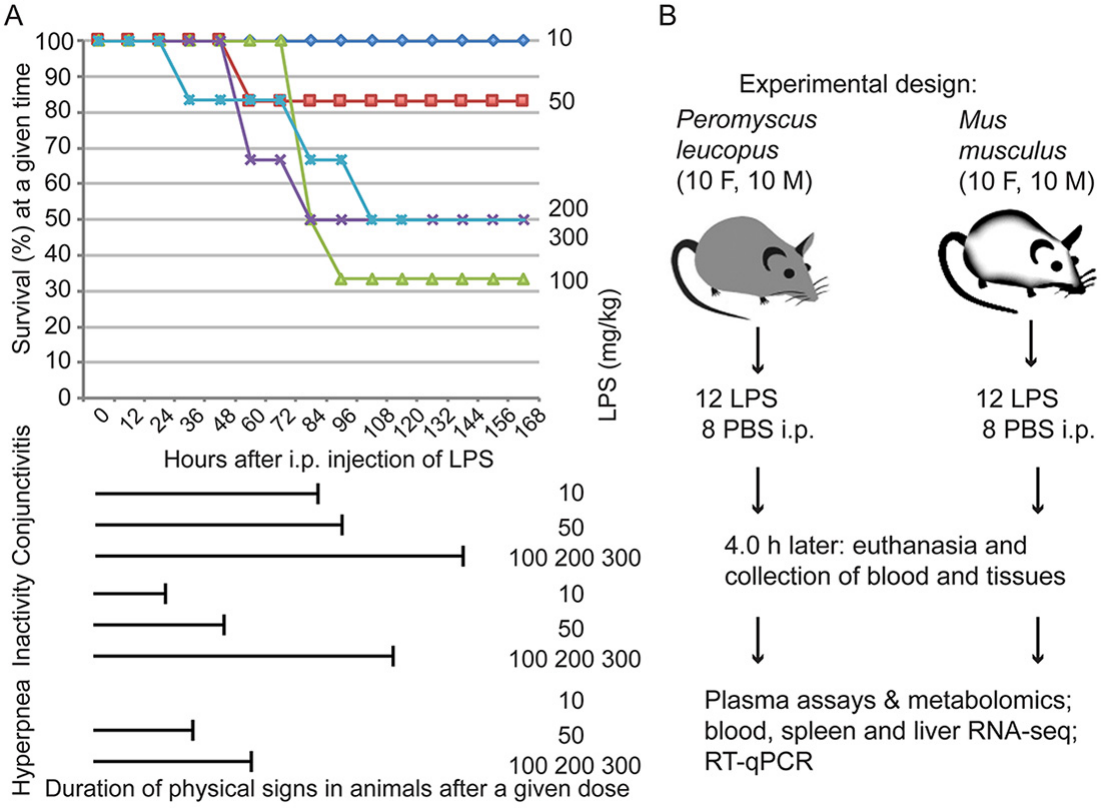
Studies of lipopolysaccharide (LPS) effects on Peromyscus leucopus deermice and experimental design. (A) Dose response of *P. leucopus* to LPS. Groups of 6 adult animals (3 females and 3 males) received different intraperitoneal (i.p.) doses on a milligram/kilogram of body weight basis of Escherichia coli LPS at time zero and then monitored for physical signs of sickness (conjunctivitis, inactivity, and hyperpnea) and survival over the succeeding 7 days (168 h). The survival curves by dose are indicated by text to the right and by colors: dark blue (10 mg/kg LPS), red (50 mg/kg), light green (100 mg/kg), blue green (200 mg/kg), and purple (300 mg/kg). The *x* axis scale and label apply to the durations of physical signs as well as to the survival graph. (B) Experimental design of comparative study of short-term effects of LPS on *P. leucopus* and Mus musculus. Animals received 10 mg LPS in saline/kg body weight or saline alone. F, female; M, male.

We began with a study of the effect of single doses of purified Escherichia coli LPS administered i.p. to adult *P. leucopus* animals and then monitored them for 7 days. Figure 1A includes the survival curve for animals in groups of six receiving doses of 10, 50, 100, 200, or 300 mg per kg and examined continuously for the first 8 h and then at 12-h intervals thereafter. Death or a moribund state occurred in at least one animal in all dosage groups except the 10-mg/kg group. Five of 6 in the 50-mg dose group survived. At higher doses, the fatality rate was higher, with death occurring between days 2 and 5. Remarkably, 3 of 6 of the animals receiving the highest dose of 300 mg per kg, or a total dose of 6 mg on average per animal, survived. Survivors among the *P. leucopus* animals at that and the 100- and 200-mg/kg doses appeared to have fully recovered by 7 days after the injection. The variation between individual animals in outcome at the higher doses was consistent with the genetic heterogeneity of this outbred population (34). From these data, we could not calculate a precise LD_50_, but we estimated it to between 100 and 300 mg/kg from this experiment.

The deermice in this experiment had an exudative conjunctivitis with eyelid closure at all doses by 12 h, and that persisted for up to 6 days at the highest dose (Fig. 1A). All animals displayed reduced activity, as defined by the behavior of huddling in groups, moving only for eating and drinking. The durations of this sign, as well as for the sign of hyperpnea, or rapid breathing, in animals receiving doses of 50 mg/kg or higher, correlated with the dose amounts. Thus, even at the lowest dose, all the animals displayed ill effects of this treatment. But in comparison to reported findings in *M. musculus*, a substantial proportion of deermice receiving doses that were 10- to 20-fold higher than the consensus LD_50_ for the house mouse did not further deteriorate and thereafter recovered.

### Experimental design.

We compared the short-term responses to a single dose of LPS of the outbred *P. leucopus* mice and the inbred *M. musculus* BALB/c strain mice of both sexes. The comparison of animals from a heterogeneous closed colony with an inbred population provided a gauge of the diversity of responses among the deermice. Figure 1B summarizes the experimental design, and Table A1 in the appendix lists the characteristics of the animals in the experiment and selected parameters. Fecal samples obtained from animals a day before the experiment had been subjected to gut microbiome analysis (35), and alpha-diversity values of the microbiota from the earlier study are provided in the table. The animals of each species were sexually mature adults and comparable in size and age, though *P. leucopus* tended to be smaller and older by 1 to 2 weeks. With the exception of two siblings split between treatment and control groups (Table A1), the *P. leucopus* mice were the offspring of different mating pairs. We alternated the administration of the LPS by species and by sex within a species to control for diurnal effects. Animals were euthanized in the same order, and the intervals between animals were kept within a strict limit. The blood cells and tissues of spleen and liver were subjected to RNA-seq, and the plasma was used for metabolomics. This comparative study was complemented by experiments with an older set of *P. leucopus* mice similarly exposed to LPS, deermice with a systemic bacterial infection, and cultures of *P. leucopus* fibroblasts.

### Comparison of deermice and mice in response to LPS.

Both species displayed effects of LPS within an hour of the injection, namely, reduced activity and ruffed fur, but only among 6 LPS-treated *P. leucopus* mice, equally distributed between females and males, did we observe conjunctivitis (*P = *0.025) (Table A1). For corticosterone concentrations, the mean for *M. musculus* controls (*n* = 7) and LPS-treated mice (*n* = 12) were 77 (95% confidence interval [CI], 34 to 120) and 624 (95% CI, 580 to 667), respectively (*P < *10^−11^). Corresponding values for *P. leucopus* control (*n* = 7) and LPS-treated (*n* =12) mice were 186 (95% CI, 79 to 293) and 699 (95% CI, 670 to 727), respectively (*P < *10^−8^). There were marginally higher baseline levels of corticosterone in *P. leucopus* than in *M. musculus* mice (*P = *0.10), but they had similar levels after LPS. The assay for nitric oxide demonstrated higher levels in 11 *M. musculus* mice treated with LPS (mean, 29 [95% CI, 20 to 37]) than in 7 controls (mean, 7 [95% CI, 3 to 12]) (*P = *0.008). There was not an elevation in nine LPS-treated *P. leucopus* mice compared with values for six controls: 7 (95% CI, 3 to 11) versus 7 (95% CI, 3 to 12), respectively (*P = *0.9).

To estimate the combined contributions of abundance and transcriptional activity of white blood cells in the blood samples at the time of euthanasia, we used the numbers of RNA-seq reads matching the whole mitochondrial genomes of each species in the same analysis (Table A1). In both species, there were lower total transcriptional activities of mitochondria in LPS-treated animals than in controls at 4 h (lower by an average of 31% in mice and 23% in deermice). Distributions were similar between species for both controls and LPS-treated animals. The mean log_10_-transformed normalized values were 5.6 (95% CI, 5.5 to 5.7) for *M. musculus* and 5.7 (95% CI, 5.6 to 5.8) for *P. leucopus* controls (Mann-Whitney *P = *0.96) and 5.4 (95% CI, 5.4 to 5.5) for *M. musculus* and 5.6 (95% CI, 5.5 to 5.7) for *P. leucopus* LPS-treated animals (*P = *0.97).

### Metabolomics of plasma.

**FIG 2:**
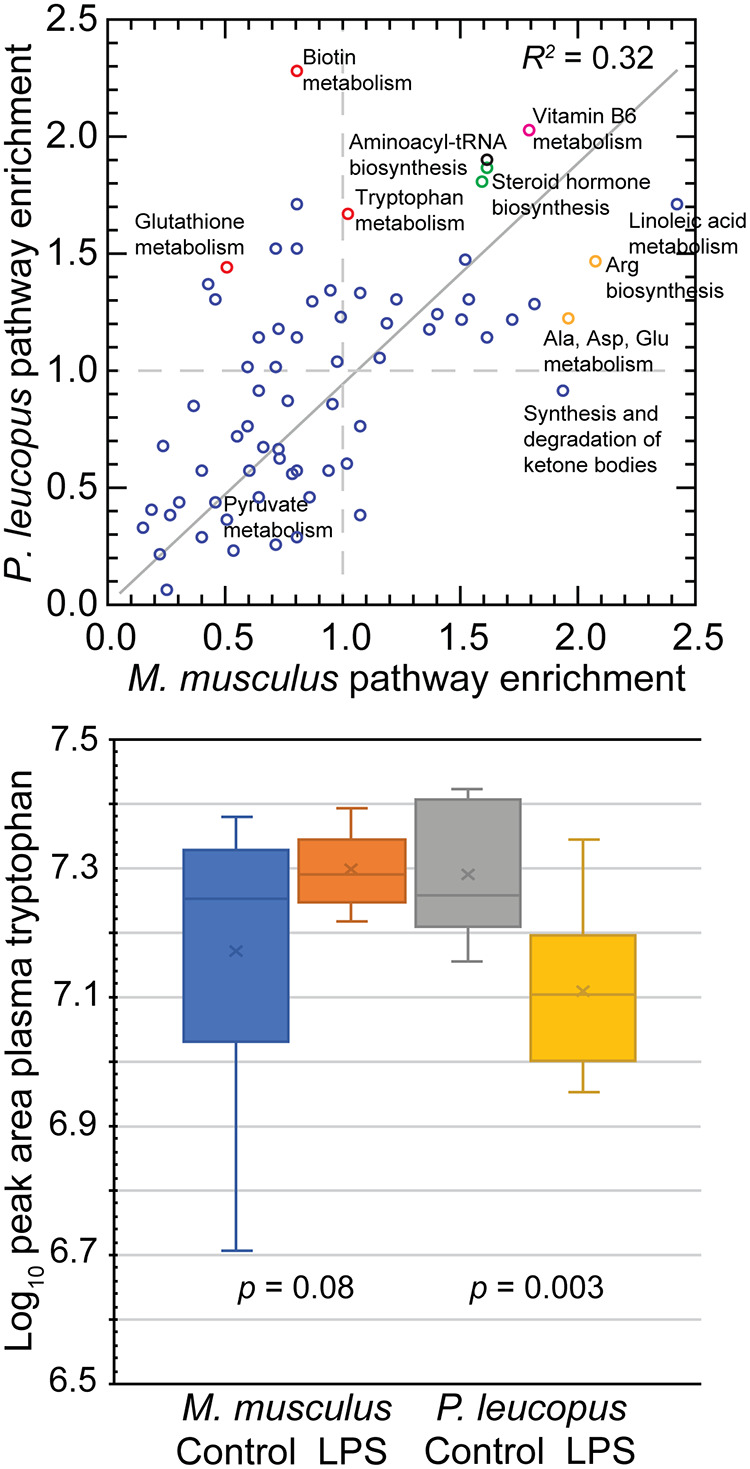
Untargeted metabolomics of plasma of *P. leucopus* and *M. musculus* animals with or without LPS treatment 4 h prior. (Top) Scatterplot of pathway enrichments in LPS-treated *P. leucopus* (*y* axis) versus those in LPS-treated *M. musculus* (*x* axis). An enrichment value of 1.0 means that there is no difference in number of compounds in a given pathway between treated and untreated conditions for a species. Data, including identified KEGG terms, are provided in the Dryad repository (https://doi.org/10.7280/D1R70J). Selected pathways are labeled. The color of the symbols indicate the following findings for false discovery rate (FDR) *P* values of <0.05: green, both species; red, *P. leucopus*; and orange, *M. musculus*. The coefficient of determination (*R^2^*) shown is for an unspecified intercept. For consistency with the dashed lines indicating enrichment values of 1.0, the regression line for an intercept of 0.0 is shown. (Bottom) Box-plots of log-transformed plasma tryptophan levels estimated as peak areas in LPS-treated and untreated animals of each species. Two-tailed *t* test *P* values between the two conditions for each species are shown. Data for tryptophan and several of its metabolites are given in Table S1.

Untargeted metabolomics identified in *M. musculus* plasma 8,125 molecular features (MF), of which 123 (1.5%) differed between LPS-treated and control animals, with a false-discovery rate (FDR) of <0.05 and an absolute fold change of >2.0. In *P. leucopus* plasma, 7,714 MF were identified, of which 215 (2.8%) correspondingly differed between treated and control animals. Pathway enrichment analysis allowed cross-species comparison of identifiable metabolites in the animals (Dryad repository, https://doi.org/10.7280/D1R70J). For *M. musculus* and *P. leucopus*, the numbers of identified pathways with one or more KEGG-defined compounds were 76 and 73, respectively, with 73 in common (Fig. 2A). In both species, there was enrichment of the steroid hormone biosynthesis pathway 4 h after LPS injection, which was consistent with the results of plasma corticosterone assays.

There was overall enrichment of tryptophan metabolism in LPS-treated animals of both species. The magnitude was greatest for *P. leucopus*, which had 30 significant hits out of a possible 41 compounds in the pathway (Fig. 2A). Tryptophan itself was significantly lower in abundance in plasma of LPS-treated *P. leucopus* animals than in untreated animals, while in *M. musculus*, it was marginally higher after LPS treatment than in controls (Fig. 2B; Table S1). Tryptophan depletion activates the amino acid sensor GCN2, which leads to increased production of IL-10 and transforming growth factor beta (TGF-β) by macrophages and dendritic cells (52). Kynurenine, the product of indole 2,3-dioxygenase (Ido1), was elevated in the LPS-treated animals over controls in both species, but the kynurenine/tryptophan ratio was higher, at 1.75, in the LPS-treated deermice than in controls but lower, at 0.79, in the LPS-treated mice (*P = *0.004) (Fig. S2; Table S1). Higher kynurenine/tryptophan ratios are associated with anti-inflammatory effects through kynurenine’s binding to the aryl hydrocarbon receptor that drives regulatory T cell differentiation (53).

10.1128/mBio.00588-21.2 FIG S2 DEGs of RNA-seq with a limited data set of orthologous protein coding sequences of *P. leucopus* and *M. musculus* for blood, spleen, and liver. (A, B, and C) Box plots for blood (A), spleen (B), and liver (C) and for representative genes. The *x* axes indicate the four different experiment groups: 8 *M. musculus* control (MC) mice, 12 *M. musculus* LPS-treated (ML) mice, 8 *P. leucopus* control (PC) deermice, and 12 *P. leucopus* LPS-treated (PL) deermice. The *y* axes are numbers of log_10_-transformed, normalized unique reads per coding sequence. (B) Spleen. Two different genes are included in the same graph when their ranges of values across both species and conditions were commensurate. (C) Liver. The box plot for Gdf15 at the bottom of the panel groups animals by sex as well as by species and treatment. Data values by individual coding sequence are given in Table S5. The accession numbers for the coding sequences are in Table A2. Download FIG S2, PDF file, 1.4 MB. Copyright © 2021 Balderrama-Gutierrez et al. 2021 Balderrama-Gutierrez et al. https://creativecommons.org/licenses/by/4.0/ This content is distributed under the terms of the Creative Commons Attribution 4.0 International license.

10.1128/mBio.00588-21.4 TABLE S1 Tryptophan metabolites in the plasma of *P. leucopus* and *M. musculus* animals with or without (control) LPS treatment as log_10_-transformed peak areas and kynurenine/tryptophan ratios for Fig. S1. Download Table S1, XLSX file, 0.02 MB. Copyright © 2021 Balderrama-Gutierrez et al. 2021 Balderrama-Gutierrez et al. https://creativecommons.org/licenses/by/4.0/ This content is distributed under the terms of the Creative Commons Attribution 4.0 International license.

10.1128/mBio.00588-21.1 FIG S1 Box whisker plots of tryptophan metabolites in plasma of *M. musculus* and *P. leucopus* 4 h after intraperitoneal injection of LPS at a dose of 10 mg/kg body weight or saline alone. The abundances as estimated from peak areas of liquid chromatography (LC)-MS are indicated on the *y* axis. Download FIG S1, PDF file, 0.4 MB. Copyright © 2021 Balderrama-Gutierrez et al. 2021 Balderrama-Gutierrez et al. https://creativecommons.org/licenses/by/4.0/ This content is distributed under the terms of the Creative Commons Attribution 4.0 International license.

### DEGs in each species.

**FIG 3:**
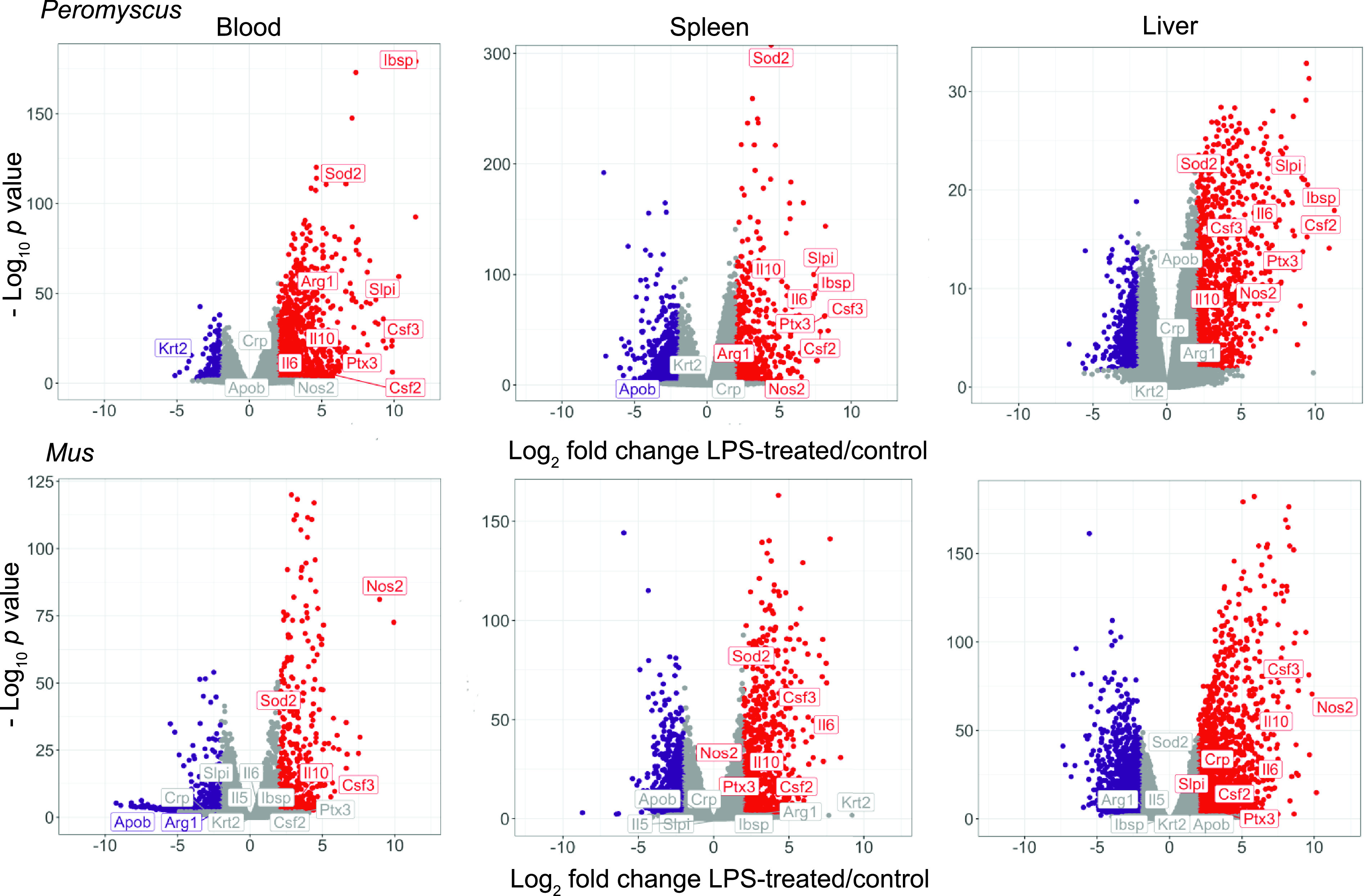
Species- and tissue-specific responses to LPS. Independent differential gene expression analysis of RNA-seq data were performed for blood, spleen, and liver tissues of *P. leucopus* and *M. musculus* collected 4 h after injection with LPS or buffer alone as a control. These are represented as volcano plots with range-adjusted scales for the log_2_-transformed fold changes on *x* axes and log_10_-transformed FDR *P* values on *y* axes. Colors of symbols denote the following: red, upregulated gene with an absolute fold change of >4.0 and a *P* value of <0.05; purple, downregulated gene with an absolute fold change of >4.0 and a *P* value of <0.05; and gray, all others. Numbers at the top left and right corners in each plot represent numbers of down- and upregulated genes, respectively. Numerical values for each gene in the 6 data sets are provided at the Dryad repository (https://doi.org/10.7280/D1VX0C).

We performed RNA-seq on blood, spleen, and liver extracts and then differential gene expression using 24,295 and 35,805 annotated transcripts for *P. leucopus* or *M. musculus*, respectively, as separate reference sets (Dryad repository, https://doi.org/10.7280/D1VX0C). Figure 3 reveals hundreds of genes that were either up- or downregulated in the blood and two organs of each species by the criteria of a ≥ 4-fold change and an FDR of <0.05. With the exception of the spleens of *P. leucopus* animals, there were more upregulated than there were downregulated genes in each of the comparisons. The highest numbers of differentially expressed genes (DEGs) were in the livers: 1,553 for *P. leucopus* and 3,250 for *M. musculus* animals.

A notable difference in the responses of *P. leucopus* and *M. musculus* was consistent with the results of the nitric oxide assays of the plasma of these animals. Inducible nitric oxide synthase or nitric oxide synthase 2 (Nos2) transcript levels were a mean of 493 times higher in the blood of LPS-treated mice than in the controls (*P = *10^−78^). But in the blood of deermice, Nos2 expression was barely detectable, and expression of Nos2 was indistinguishable between the two conditions (*P = *0.34). In contrast, arginase 1 (Arg1), which by its action reduces the amount of arginine available for Nos2 to produce nitric oxide, was 21 times higher in expression in the blood of LPS-treated *P. leucopus* animals than in controls (*P = *10^−47^), while in *M. musculus* blood, Arg1 expression 4 h after LPS injection was 6 times lower than baseline expression (*P = *0.04). This reciprocal expression profile for Nos2 and Arg1 between the two species was also observed in the spleen. The products of Nos2 and Arg1 are informative biomarkers for categorizing polarized macrophage responses (54).

Two other genes whose expression profiles distinguished the species in LPS responses were the genes for integrin-binding sialoprotein (Ibsp) and secretory leukocyte peptidase inhibitor (Slpi). These were first and fourth ranked of the upregulated DEGs in the blood for *P. leucopus*, but they ranked numbers 6,232 and 33,541, respectively, among measured transcripts in the blood for *M. musculus* (Dryad repository https://doi.org/10.7280/D1VX0C). The fold differences between Ibsp and Slpi in the LPS-treated deermice and control animals were 2,903 times (*P = *10^−174^) and 1,280 times (*P = *10^−57^) higher, respectively. In contrast, Ibsp was slightly expressed in mice under both conditions (the number of transcripts per million [TPM] was 0.001), and Slpi in expression was lower by 3× in the treated mice (FDR *P = *0.04).

### Sex-specific responses to LPS in *P. leucopus*.

To identify sex-specific responses, the DEG analysis was applied to the RNA-seq of the blood and spleens of *P. leucopus* animals treated with LPS or saline alone (Table S2). A noncoding RNA (GenBank accession no. XR_003736827) was expressed at orders-of-magnitude-higher levels in females than in males in all tissues. This noncoding RNA (ncRNA) was revealed as the inactive X-specific transcript (Xist), which functions to inactivate genes of one X chromosome of females (55). D1Pas1, an autosomal DEAD box RNA helicase, which is expressed in the testis in mice (56), was markedly lower in expression in all females than in males. Two genes that were expressed by a thousandfold more abundantly in the blood of LPS-treated females than in both control females and the males were those encoding adiponectin receptor 2 (Adipor2) and the X-linked lysine-specific demethylase 6A (Kdm6a). Kdm6a has been implicated in the risk of acquiring autoimmunity and reported to regulate multiple immune response genes (57). Adiponectin, an adipokine, has an anti-inflammatory effect (58), and expression of its receptor is reportedly affected by the macrophage polarization phenotype (59). Adipor2 was also highly expressed in the spleens of LPS-treated females.

10.1128/mBio.00588-21.5 TABLE S2 Genes differentially expressed after LPS treatment between female and male *P. leucopus* animals in blood and spleen. Download Table S2, XLSX file, 0.02 MB. Copyright © 2021 Balderrama-Gutierrez et al. 2021 Balderrama-Gutierrez et al. https://creativecommons.org/licenses/by/4.0/ This content is distributed under the terms of the Creative Commons Attribution 4.0 International license.

### Functional processes distinguishing and shared between species.

**FIG 4:**
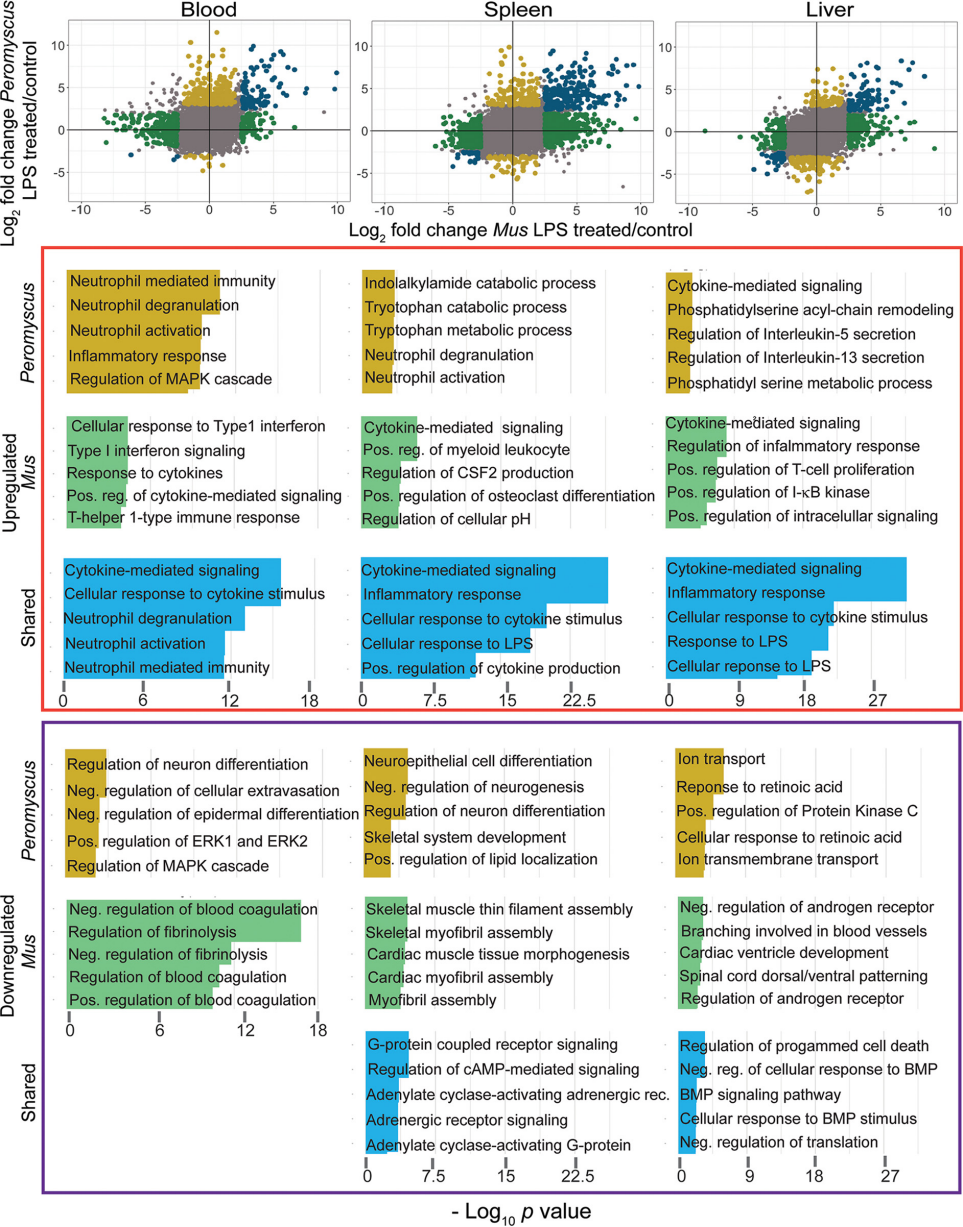
Comparison of *P. leucopus* and *M. musculus* animals in their responses to LPS by RNA-seq and categorization of DEGs by gene ontology (GO) term enrichment. In the three scatterplots for blood, spleen, and liver tissues at the top of the figure, log_2_ values for the fold change of *P. leucopus* are plotted against corresponding values for *M. musculus* for each gene in the data set. DEGs specific for *P. leucopus* are indicated by gold symbols, while DEGs specific for *M. musculus* are green. Genes shared between species among the DEGs are in blue. Gray is for all others. *P. leucopus*-specific, *M. musculus*-specific, and shared upregulated genes are in the upper half, right half, or upper right quadrant, respectively, of the plot. GO term enrichment was performed for each group of genes, separating upregulated and downregulated genes for each one of the tissues, accordingly. The colors in the horizontal bar graphs correspond with the colors indicated above. The genes that constitute each of the listed GO terms in the bottom part of the figure are given in Table S3. Pos., positive; reg., regulation; Neg., negative; MAPK, mitogen-activated protein kinase; cAMP, cyclic AMP; rec., receptor.

To further define similarities and difference between the responses of the two species to LPS, we identified gene groups categorized by gene ontology (GO) terms as *P. leucopus* specific, *M. musculus* specific, or shared, meaning, within each pairing, the enrichment groups that were up- or downregulated. For this analysis, 14,685 one-to-one orthologues were used for the comparisons. Figure 4 summarizes this analysis for blood, spleen, and liver. The genes associated with each GO term for which there was significant enrichment are listed in Table S3.

10.1128/mBio.00588-21.6 TABLE S3 Enrichment GO terms and included genes for Fig. 4. Download Table S3, XLSX file, 0.03 MB. Copyright © 2021 Balderrama-Gutierrez et al. 2021 Balderrama-Gutierrez et al. https://creativecommons.org/licenses/by/4.0/ This content is distributed under the terms of the Creative Commons Attribution 4.0 International license.

In the blood, both species showed upregulation of some components of cytokine-mediated signaling and cellular responses to cytokines. For cytokine-associated genes, these included the aconitate decarboxylase 1 (Acod1), chemokine C-X-C motif ligand 10 (Cxcl10), Cxcl11, Il1a, Il10, interleukin 1 receptor (Il1r), interleukin 1 receptor antagonist (Il1rn), superoxide dismutase 2 (Sod2), and TNF genes. Neutrophil-associated genes upregulated in the blood of both species included those for CD14 antigen (Cd14), Cd177, formyl peptide receptor 1 (Fpr1), Fpr2, lipocalin 2 (Lcn2), matrix metallopeptidase 8 (Mmp8), Ptx3, and Toll-like receptor 2 (Tlr2). The *Peromyscus*-specific upregulated profile in the blood featured an expanded set of genes constituting GO terms for neutrophil activities, including arachidonate 5-lipoxygenase (Alox5), Cd33, C-X-C motif receptor 2 (Cxcr2), leucine-rich alpha-2 glycoprotein (Lrg), Mmp9, resistin (Retn), and Slpi. The blood samples of *M. musculus* uniquely featured GO terms related to (i) blood coagulation and fibrinolysis, including apolipoprotein H (Apoh), coagulation factor II (F2), coagulation factor XII (F12), plasminogen (Plg), and protein C (Proc), and (ii) type 1 interferon signaling and cellular responses, including signal transducer and activator of transcription 2 (Stat2) and XIAP-associated factor 1 (Xaf1).

There were 297 DEGs for the spleen that were shared between *Peromyscus* and *Mus* (Table S3), but there were distinguishing features as well. Up-regulated GO terms for the *P. leucopus* spleen were tryptophan metabolic and catabolic processes, specifically Ido1. For *M. musculus*, uniquely associated GO terms that were upregulated in the spleen dealt with production and differentiation of myeloid cells, macrophages, and osteoclasts and included the cytokines IL-12 (Il12b) and interleukin 17 members IL-17A (Il17a), Il17f, and IL-23 (Il23a).

In the liver samples, four upregulated GO terms that distinguished the deermice from the mice were phosphatidylserine metabolic process and phosphatidylserine acyl-chain remodeling, which included phospholipase A2, group IIA (Pla2g2a), Pla2g5, and Pla1a; regulation of IL-5 and IL-13 secretion, both of which included the transcription factor GATA binding protein 3 (Gata3) (60); and tumor necrosis factor receptor superfamily, member 21 (Tnfrsf21), which reportedly is a determinant of influenza A virus susceptibility in mice (61). Two distinguishing GO terms for downregulated genes in the livers of LPS-treated *P. leucopus* animals were response to retinoic acid and cellular response to retinoic acid.

A distinguishing GO term for upregulated genes in *M. musculus* liver was positive regulation of I-kB kinase/NF-κB signaling, which included the NLR family, apoptosis inhibitory protein 5 or Naip5 (Birc), Ccl19, inhibitor of kappaB kinase epsilon (Ikbke), Il12, Il1α, LPS-induced TN factor (Litaf), Myd88, receptor tyrosine-kinase orphan receptor 1 (Ror1), Tlr2, and tumor necrosis factor (ligand) superfamily member 10 (Tnfsf10). Distinguishing GO terms for downregulated genes in LPS-treated mouse liver concerned the androgen receptor signaling pathway and included the transcription factor forkhead box H1 (Foxh1) and hairy/enhancer-of-split related with YRPW motif-like (Heyl), which is associated with repression of TGF-β signaling (62).

In summary, this layering of established GO terms over the DEG analysis provided consolidation and further evidence that *P. leucopus* and *M. musculus* have much in common in how they respond to LPS in the blood, spleen, and liver in the first few hours. But there were also several distinguishing functional processes. One feature of the *P. leucopus* response that particularly stood out in this analysis was the activation of neutrophils and other phagocytes. Included in the lists of specific genes under all the neutrophil-associated GO terms was the gene for Slpi, which was first identified as an inhibitor of serine proteases, such as neutrophil elastase (63). In *M. musculus*, notable distinguishing GO terms concerned blood coagulation and fibrinolysis, processes that figure in the pathophysiology of sepsis (64).

### Gene network patterns across species.

**FIG 5:**
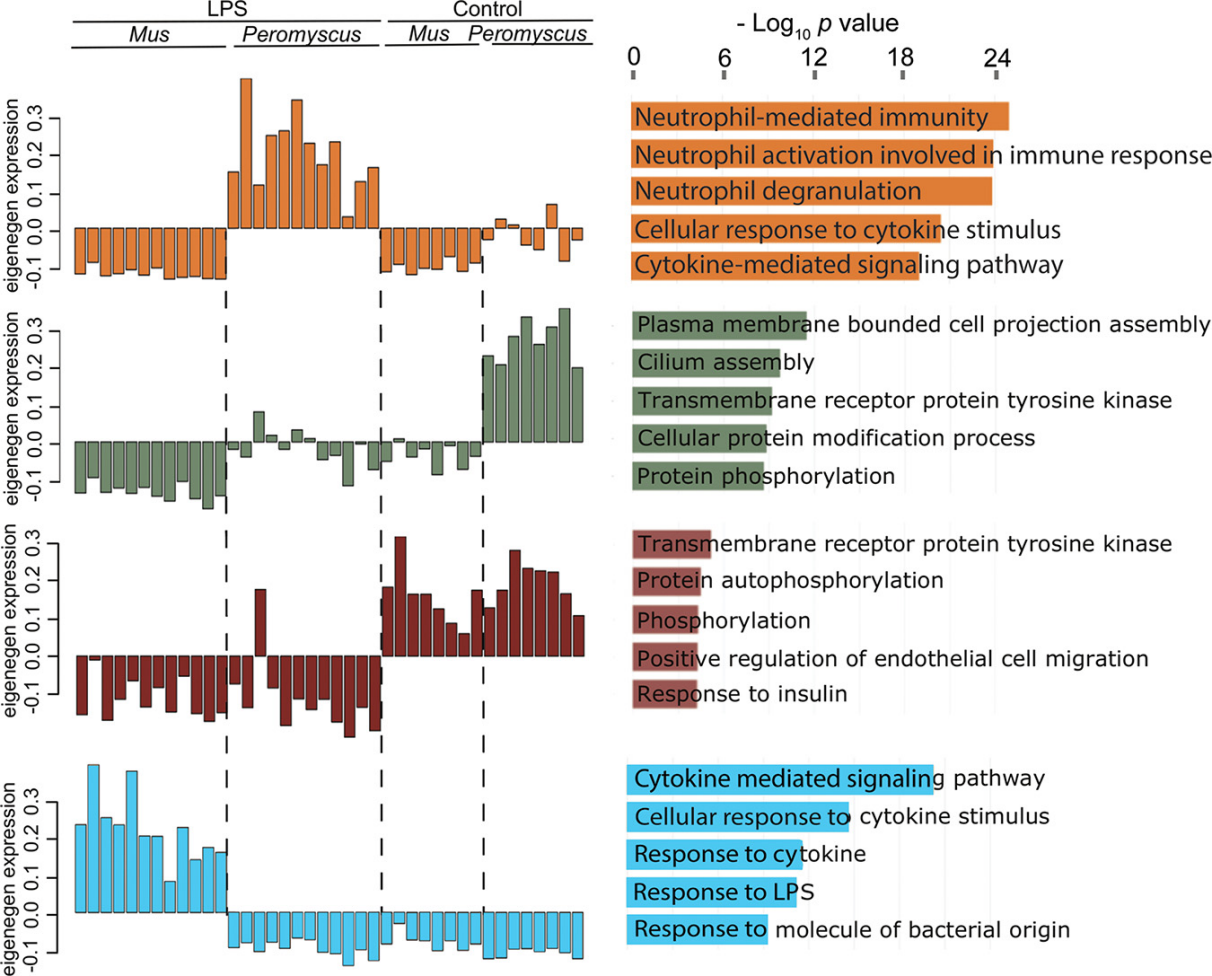
Four selected Eigengene modules by network analysis of differential responses of *P. leucopus* or *M. musculus* animals to LPS. The different modules are distinguished by the color of the hexadecimal scheme: dark orange 2 for upregulated in LPS-treated *P. leucopus*, dark sea green 4 for comparatively higher expression in untreated *P. leucopus* than in the other three groups, brown 4 for downregulated in both species after LPS treatment, and light blue for upregulated in LPS-treated *M. musculus*. The top 5 GO terms by adjusted *P* value are shown for each module. The DEGs constituting each of the GO term sets from this analysis are listed in Table S4 along with *P* values and odds ratios. The other 20 modules from this analysis are available at the Dryad repository (https://doi.org/10.7280/D1B38G).

To further delineate the gene expression networks without a prior categorization, we performed weighted gene correlation network analysis (WGCNA) to empirically identify groups of genes (modules) that distinguished specific species and/or treatments in response to LPS. Gene network analysis allowed identification of patterns across the three tissues. Since blood and the two organs inherently differed in their expression profiles across the genome and one tissue may overshadow the patterns in other tissues, we made a matrix in which each column represented a sample and each row represented a gene per tissue. Each gene had a suffix to identify the tissue of origin. Twenty-four modules were identified, and GO term enrichment was assessed for unique genes for each module. Figure 5 summarizes the results for four modules: darkorange2, darkseagreen3, brown4, and lightblue. The associated GO terms and constituent genes of the four modules are listed in Table S4.

10.1128/mBio.00588-21.7 TABLE S4 Enrichment GO terms and included genes of the four modules of Fig. 5. Download Table S4, XLSX file, 0.03 MB. Copyright © 2021 Balderrama-Gutierrez et al. 2021 Balderrama-Gutierrez et al. https://creativecommons.org/licenses/by/4.0/ This content is distributed under the terms of the Creative Commons Attribution 4.0 International license.

The darkorange module comprised genes that were upregulated in LPS-treated *P. leucopus* and not in either control *P. leucopus* or LPS-treated or control *M. musculus*. The blood made the largest contribution, with 1,472 genes to this module, followed by liver and spleen, with 933 and 776, respectively, each. The highest three ranked GO terms related to neutrophils: neutrophil-mediated immunity, neutrophil activation involved in immune response, and neutrophil degranulation. The three GO terms share several neutrophil-associated genes that were upregulated, including those for lysosomal cysteine proteases cathepsin S (Ctss) and cathepsin B (Ctsb) and lysosomal membrane protein 2 (Lamp2). The Slpi gene was also among the contributing genes for each of the three neutrophil GO terms.

The lightblue module comprised genes that were upregulated in LPS-treated *M. musculus* but not in either control mice or LPS-treated or control *P. leucopus* animals. In contrast to the darkorange module, the liver made the largest contribution to the lightblue module, with 1,170 genes, followed by spleen, with 566 genes, and blood, with 33 genes. The three highest-ranked GO terms for this module were cytokine-associated: cytokine-mediated signaling pathway, cellular response to cytokine stimulus, and response to cytokine. While the darkorange module also included two cytokine-associated GO terms at a lower rank than the three neutrophil-associated GO terms, only a minority of genes (41 of 170) in the combined list overlapped between the two species for the cytokine-mediated signaling pathway GO term. Selected upregulated genes unique to LPS-treated *M. musculus* under this GO term included those for the following: cytokines Il1a, Il12, Il17, Il22, Il27, Il33, and Ifng; the chemokines Ccl5 (RANTES), Cxcl9, and Cxcl13; macrophage markers Cd86 and Cd80; and transcription factors Jak3 and Stat4. Selected DEGs associated with LPS-treated *P. leucopus* for this GO term included those for the following: Alox5, annexin 1 (Anxa1), Casp3, leptin (Lep), Sod2, Tgfb1, and tissue inhibitor of metalloproteinases (Timp1); the cytokines Il1b, Il10, Il19, and interferon beta (Ifn1b); and the transcription factors Gata3, Irf8, Stat1, and Stat3.

The two other modules highlighted in Fig. 5 and Table S4 are the darkseagreen module, which distinguished control *P. leucopus* from the other 3 groups, and the brown module, which featured GO terms that were downregulated in comparison to controls in both sets of LPS-treated animals. For the dark sea green module, the spleen was the major contributor, with 4,791 genes, followed by liver, with 3,189 genes, and blood, with 73 genes. Two GO terms with many genes in common were plasma membrane-bounded cell projection assembly and cilium assembly. For the brown module, the liver RNA-seq with a contribution of 876 genes far out-numbered the contributions of spleen, with 6 genes, or blood, with 3 genes. Genes common to the top three GO terms in this module were all genes for kinases: tyrosine-protein kinase ABL (Abl1), tyrosine-protein kinase CSK (Csk), mitogen-activated protein kinase 3 (Mapk3), and megakaryocyte-associated tyrosine kinase (Matk).

### RT-qPCR of Nos2, Arg1, and Slpi.

In analyses of RNA-seq data at the gene, pathway, or network level, the Slpi, Nos2, and Arg1 genes were three of the genes that distinguished the species. We confirmed the markedly higher expression of Slpi in *P. leucopus* blood after LPS by reverse transcriptase quantitative PCR (RT-qPCR) of replicate cDNA libraries from the RNA extracts (Table 1). The assays also confirmed the absent-to-low expression of Nos2 in *P. leucopus* blood in both LPS-treated and control animals, as well as the high baseline expression of Arg1 in the deermice and even higher in the LPS-exposed animals (Table 1).

**TABLE 1:** RT-qPCR of selected transcripts in blood of *P. leucopus* animals with and without LPS treatment

mRNA	Control (*n* = 8) mean no. of copies (95% CI)	LPS (*n* = 12) mean no. of copies (95% CI)	*t* test *P* value
Glyceraldehyde 3-phosphate dehydrogenase (Gapdh)	55,888 (32,512–96,070)	135,970 (75,934–243,471)	>0.05
Nitric oxide synthase 2 (Nos2)	0 (0–0)	5 (1–9)	>0.05
Arginase 1 (Arg1)	310 (199–481)	8,779 (5,185–14,864)	4 × 10^−8^
Secretory leukocyte peptidase inhibitor (Slpi)	5 (3–9)	43,524 (29,039–65,235)	3 × 10^−16^

### Comparative expression of other genes.

**FIG 6:**
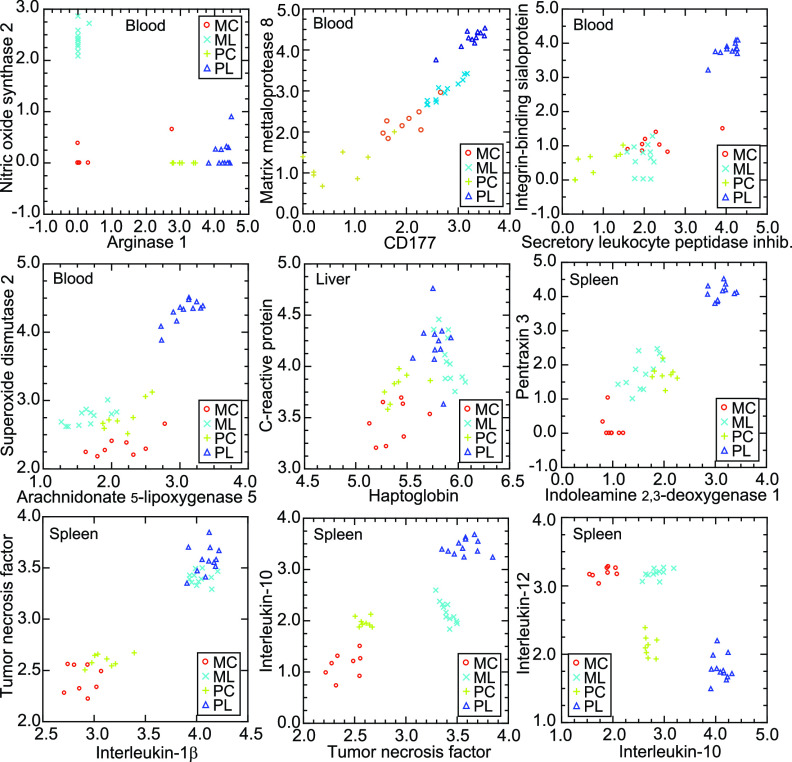
Correlations of pairs of selected genes of *P. leucopus* and *M. musculus* from the RNA-seq analysis of Table S3 and Fig. S2. The 9 scatterplots are log_10_ values of normalized unique reads of one coding sequence against another for each of the four groups: control *M. musculus* (MC), LPS-treated *M. musculus* (ML), control *P. leucopus* (PC), and LPS-treated *P. leucopus* (PL). Each group is represented by a different symbol.

We examined other selected coding sequences in a cross-species analysis. Those chosen either typified one of the species’ responses to LPS or were shared between species. Some genes were identified through gene-level DEG analyses (Fig. 3). Others were highlighted by GO term analysis (Fig. 4) or module-based analysis (Fig. 5). There was a single reference set that contained the pairs of orthologous protein coding sequences (CDS) of the mRNAs for both *P. leucopus* and *M. musculus*. For each of the 40 animals, there were 15 genes for blood, 40 genes for spleen, and 12 genes for liver samples (Table S5). Density distributions of the individual values in the each of the four experimental groups were plotted for 11 genes in blood, 12 genes in spleen, and 9 genes in liver (Fig. S2). Figure 6 consolidates subsets of these as plots of 9 pairs of genes and presents results for blood, spleen, and liver.

10.1128/mBio.00588-21.8 TABLE S5 RNA-seq of blood, spleen, and liver of *P. leucopus* and *M. musculus* animals with or without LPS and presented as numbers of log_10_-normalized unique reads. Download Table S5, XLSX file, 0.06 MB. Copyright © 2021 Balderrama-Gutierrez et al. 2021 Balderrama-Gutierrez et al. https://creativecommons.org/licenses/by/4.0/ This content is distributed under the terms of the Creative Commons Attribution 4.0 International license.

Up to a certain point, events after exposure to LPS appear similar in both species. Responses for both deermice and mice were highly correlated between the genes for TNF and IL-1β in the spleen and between the genes for the acute-phase reactants C-reactive protein, haptoglobin, and serum amyloid A (65) (Fig. 6; Fig. S2; Table S5). A couple of representative genes that were upregulated in both species but to a much greater extent in deermice were the Ptx3 and Ido1 genes. Pentraxin 3 is a member of a pattern recognition family involved in innate responses, and IDO1 is the rate-limiting enzyme of tryptophan catabolism by the kynurenine pathway. Untreated *P. leucopus* and *M. musculus* displayed similar expression levels for the genes for the mitochondrial iron/manganese superoxide dismutase 2 (Sod2), an antioxidant, and arachidonate 5-lipoxygenase 5 (Alox5), which transforms essential fatty acids into leukotrienes. Both Alox5 and Sod2 were 10-fold higher in expression in the blood of LPS-treated deermice but about the same in all mice (Fig. 6; Fig. S2; Table S5). The difference between untreated animals and LPS-treated *P. leucopus* animals was even more striking for Slpi and Ibsp in the blood.

The previously noted discordance between the species in Nos2 and Arg1 expression was observed in this analysis as well. The profile of high Nos2-low Arg1 expression in response to LPS indicated a type M1 macrophage response in the mouse, while the low-Nos2–high-Arg1 profile in *P. leucopus* was more typical of the type M2 macrophage response. Further evidence of a dichotomy corresponding to polarized macrophage categories was the Il10 to Il12 ratio in the spleen (66) (Fig. 6 and Table S5). In control *M. musculus* animals, the mean ratio was 0.036 (95% CI, 0.026 to 0.046), rising 10-fold to 0.361 (95% CI, 0.251 to 0.471) in LPS-treated animals. The baseline Il10 to Il12 ratio of 3.47 (95% CI, 2.32 to 4.63) was a hundredfold higher in control deermice than in their mouse counterparts. For LPS-treated *P. leucopus* animals, the ratio was 191 (95% CI, 123 to 259), many hundredfold higher than in LPS-treated *M. musculus* mice.

Gauges of neutrophil or other white blood cell activity in the blood were Itgam (also known as CD11b), Fcgr1 (CD64), Fcgr2b (CD32), Cd177, and Mmp8 (Table S5; Fig. 6; Fig. S2). In mice treated with LPS, Itgam and Fcgr2b expression was either marginally lower than in controls or unchanged, while both genes were upregulated by severalfold in the LPS-treated deermice (Table S5). Fcgr1 expression was higher in treated mice than in controls but in deermice was undetectable under both conditions (Table S5). The pair Cd177 and Mmp8 provided the best discrimination between the two species (Fig. 6). Values for Cd177 and Mmp8 were tightly associated (*R^2^* = 0.87) over the entire range for both species.

One of the genes associated with responses to LPS was growth differentiation factor 15 (Gdf15), which, among its ascribed functions, mediates tissue tolerance through triglyceride metabolism (67), but this gene was not annotated in the current genome assembly for *P. leucopus*; consequently, a corresponding transcript was absent from the reference set. Using the liver RNA-seq data, we performed a targeted analysis with the *M. musculus* coding sequence for Gdf15 and, as a substitute for *P. leucopus*, the orthologue in *P. maniculatus* (Fig. S2; Table S5). The log_10_-transformed normalized reads were higher in LPS-treated mice than in controls: 2.7 (95% CI, 2.5 to 2.9) versus 1.9 (95% CI, 1.8 to 2.1) (*P = *0.0004). This is an average fold change of 5.2. For the deermice, the reads in log_10_ values for controls were nearly identical to those for control mice: 1.9 (95% CI, 1.8 to 2.1). In contrast to mice, numbers were marginally lower in LPS-treated deermice: 1.8 (95% CI, 1.7 to 1.8). The difference between the species in the Gdf15 response was largely attributable to higher values among treated male mice (Fig. S2).

### Diversity of responses within the populations.

**FIG 7:**
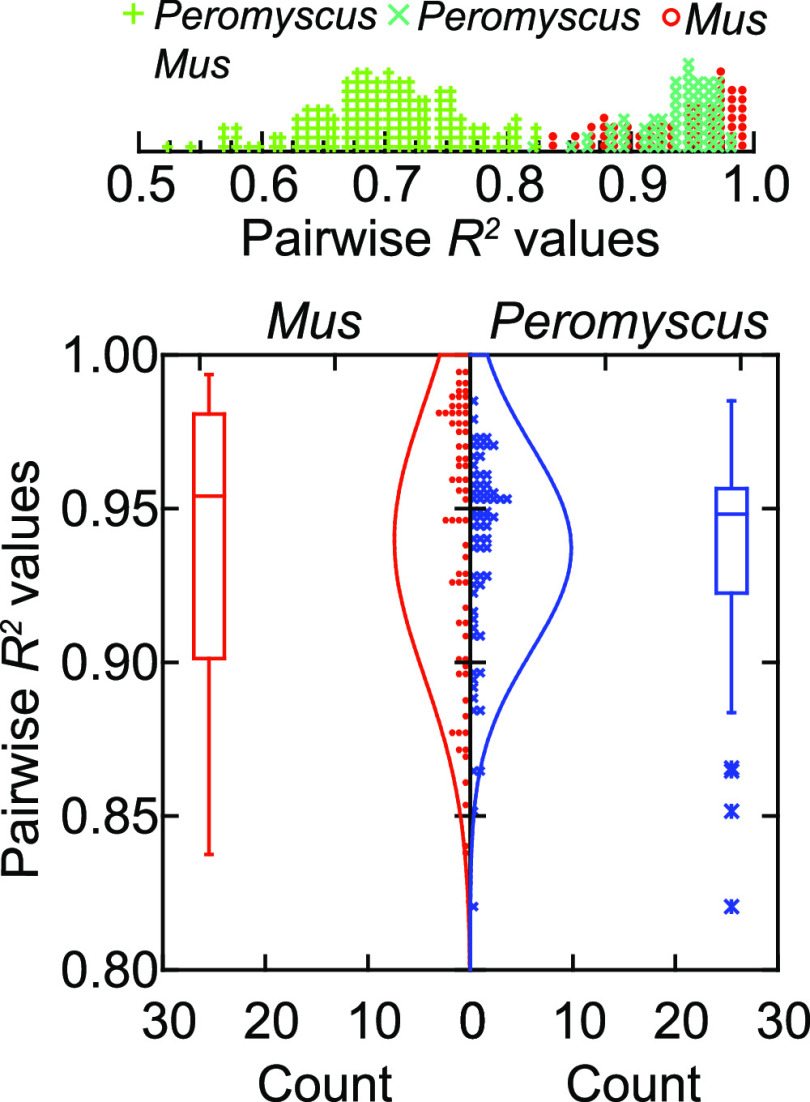
Assessment of diversity among individual animals by species in transcriptional responses to LPS for 17 genes. Pairwise coefficients of determination (*R^2^*) were calculated for the 66 intraspecies pairs for LPS-treated *P. leucopus* animals, the 66 intraspecies pairs for LPS-treated *M. musculus* animals, and the 144 interspecies pairs for all LPS-treated animals. Data were drawn from selective transcripts from RNA-seq for the spleen (Table S5). The top panel is a frequency distribution of *R^2^* values for all pairwise determinations. The bottom panel shows just the distributions of intraspecies pairwise determinations.

We investigated whether the outbred deermice were more heterogeneous in their responses than the inbred BALB/c mice with a pairwise analysis of coefficients of determination (*R^2^*) among the 24 animals that received LPS. We used spleen because of 150-nucleotide (nt) reads for this tissue and similar numbers of up- and downregulated DEGs in both species (Fig. 3). With self-pairings excluded, there were 66 pairs each for *P. leucopus* or *M. musculus* and 144 cross-species pairs. The genes were those for Alox5, Ccl2, Csf2, Csf3, Cxcl11, Gapdh (glyceraldehyde-3-phosphate dehydrogenase), Hmox1, Il1b, Il1rn, Il6, Lcn2, MT-Co1, Nfe2l2, Slpi, Sod2, Tgfb1, and Tnf, and the data were drawn from Table S5. The median fold difference between *P. leucopus* and *M. musculus* for these genes was 1.2. Figure 7 shows the distributions of *R^2^* values of the sets of pairs. As expected, correlations were lower between mixed-species pairs than between intraspecies pairs (upper panel); the median *R^2^* was 0.701. The seven highest intraspecies pairwise *R^2^* values (0.987 to 0.994) were observed among the *M. musculus* mice, and the median of the 66 *R^2^* values was marginally higher, at 0.954, for the mice than the 0.948 for the deermice, but, overall, *P. leucopus* animals in this experiment were not notably more diverse in their responses than were the inbred mice under the same conditions (*P = *0.68; Mann-Whitney *P = *0.19), an indication that the discriminating DEGs, enrichment GO terms, and modules were not wholly or partially attributable to greater variances among the deermice than the mice.

### RNA-seq of older *P. leucopus* animals in response to LPS.

We found similar profiles in blood and spleen samples of older animals in an experiment that was carried out before the deermouse-mouse comparison but under the same conditions: a 10-μg/g dose or buffer alone as a control and samples taken after 4 h. The 16 animals (12 females) had a median age of 81 weeks, with a range of 54 to 94 weeks (Table A1). Nine animals received LPS and seven buffer alone. Figure S3 comprises box plots of log-transformed normalized unique reads for pairs of genes in either the blood or the spleen of these animals. We observed again in this second experiment low baseline and after-treatment expression of Nos2 in the blood but high baseline expression of Arg1, with a further increase in the LPS-treated animals (Table S6). Both Slpi and Ibsp increased more than a hundredfold in expression in the blood after LPS. In the spleen, Il1b, Tnf, Il6, Il10, Ccl2, Cxcl11, Csf2, and Csf3 all were increased in the LPS-treated animals, as were two genes that notably were more upregulated in the samples from young *P. leucopus* deermice than in comparably aged *M. musculus* mice: the Ido1 and Ptx3 genes.

10.1128/mBio.00588-21.3 FIG S3 DEGs of RNA-seq of selected protein coding sequences for blood or spleen samples from *P. leucopus* deermice of 1 to 2 years of age. The figure comprises 9 box plots for representative genes, singly or in pairs. The *x* axes indicate the treatment groups: control or LPS treated. The *y* axes are numbers of log_10_-transformed, normalized unique reads per coding sequence. Data values by individual coding sequence are given in Table S6. Download FIG S3, PDF file, 0.5 MB. Copyright © 2021 Balderrama-Gutierrez et al. 2021 Balderrama-Gutierrez et al. https://creativecommons.org/licenses/by/4.0/ This content is distributed under the terms of the Creative Commons Attribution 4.0 International license.

10.1128/mBio.00588-21.9 TABLE S6 Limited gene set analysis of RNA-seq of spleen and blood of older *P. leucopus* animals with or without LPS treatment and presented as numbers of log_10_-normalized unique reads. Download Table S6, XLSX file, 0.02 MB. Copyright © 2021 Balderrama-Gutierrez et al. 2021 Balderrama-Gutierrez et al. https://creativecommons.org/licenses/by/4.0/ This content is distributed under the terms of the Creative Commons Attribution 4.0 International license.

### Systemic bacterial infection.

The simplicity of the LPS model of inflammation was a strength, but how representative was it of a bacterial infection in terms of the findings to this point? We chose the relapsing fever agent Borrelia hermsii, because *Peromyscus* species are natural reservoirs for the species (68), and because it has lipoproteins, instead of LPS, that are the ligands for Toll-like receptor signaling (69, 70). For this question, five *P. leucopus* were infected with the relapsing fever agent Borrelia hermsii on day 0. Another 3 animals received buffer alone. Infection of the blood was directly confirmed by microscopy on day 4. The animals were euthanized on day 5, just before the appearance of neutralizing antibody was anticipated (71). The infected animals had enlarged spleens, as well as large numbers of bacteria in the spleens, as assessed by quantitative PCR (Table S7A). We used previously collected but unanalyzed PE100 reads for the blood of these animals (33). As noted in the two LPS experiments, Nos2 expression was hardly detectable in either the controls or the infected animals. On the other hand, Arg1 expression was high at baseline and further elevated by day 5 of infection. Two genes of *P. leucopus* that clearly distinguished this species from *M. musculus* in its short-term response to LPS were the Slpi and Ibsp genes; both of these were a hundredfold more highly expressed in infected animals than in controls.

10.1128/mBio.00588-21.10 TABLE S7 Systemic infection of *P. leucopus* animals with Borrelia hermsii MTW (part A) and log_2_ fold changes of DEGs of blood of LPS-treated *P. leucopus* and Borrelia hermsii-infected *P. leucopus* animals (part B). Download Table S7, XLSX file, 0.1 MB. Copyright © 2021 Balderrama-Gutierrez et al. 2021 Balderrama-Gutierrez et al. https://creativecommons.org/licenses/by/4.0/ This content is distributed under the terms of the Creative Commons Attribution 4.0 International license.

**FIG 8:**
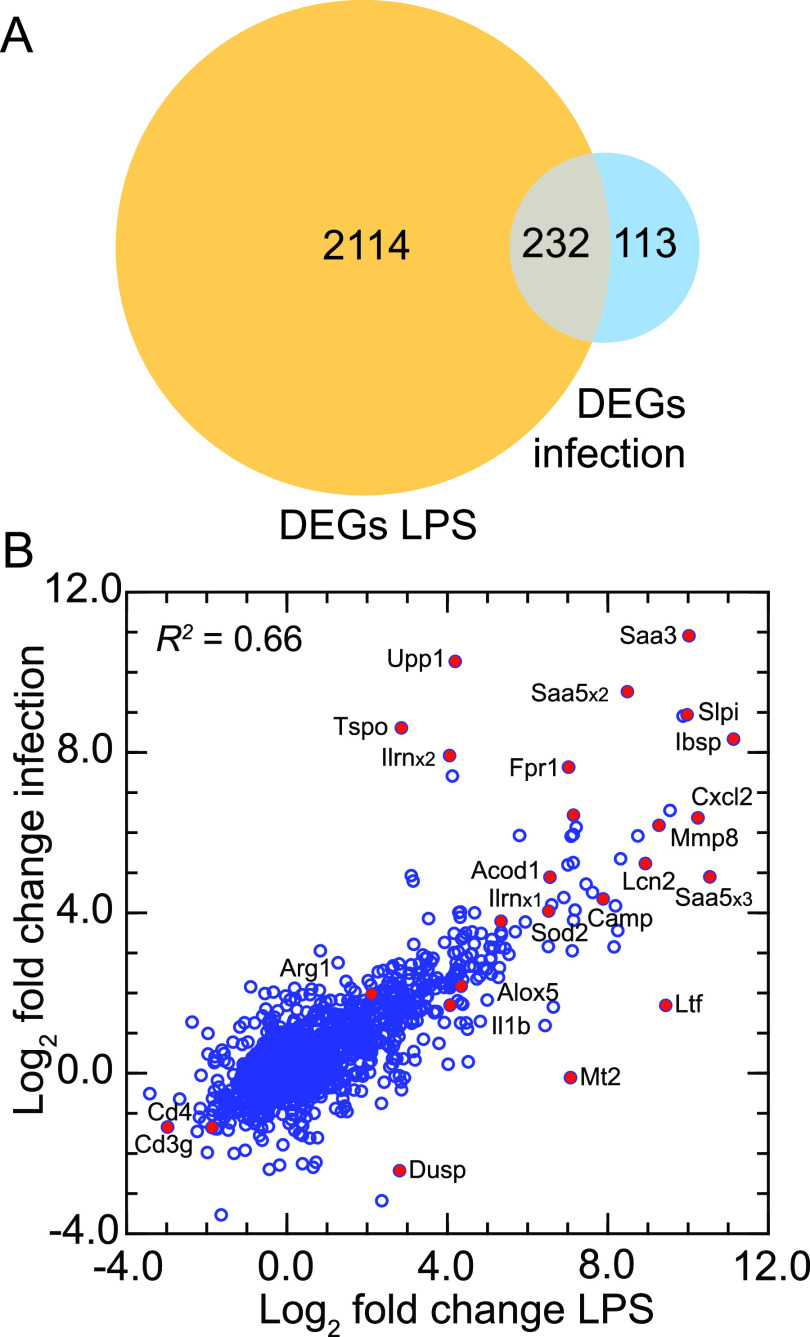
Comparison of DEGs of RNA-seq of *P. leucopus* deermice treated with LPS and *P. leucopus* animals systemically infected with the bacterial agent Borrelia hermsii. There were 12 LPS-treated animals with 8 controls and 5 infected animals with 3 controls (Table S7). (A) Venn diagram of numbers of DEGs in each experiment and the overlap between them (B) scatterplot of log_2_-transformed fold changes between study and control conditions for the infection experiment (*y* axis) versus the short-term LPS experiment (*x* axis). Selected genes are indicated with a label adjacent to a red symbol for the data point.

RNA-seq was carried out with the same reference set and settings as for the samples in a comparative response study. Given the smaller sample size of the infection study, we focused on more highly expressed genes. Of the 46,154 transcripts in the full reference set, 1,773 had mean TPM values of ≥50 in at least one of the two groups in each experiment. The latter set was used as the basis for determining and then comparing fold changes between the LPS condition with a duration in hours and the systemic bacterial infection condition with a duration in days (Table S7B). Figure 8 summarizes the pairwise fold changes for individual genes. Two-thirds of the DEGs in the infection experiment were also represented among the DEGs in the LPS experiment. We noted again the substantial upregulation of Slpi and Ibsp. Other coding sequences upregulated under both conditions were Acod1, Alox5, Arg1, Cxcl2, Il1rn, Il1b, Lcn2, and Mmp8. A discordant DEG was dual-specificity phosphatase (Dusp), which has been implicated in regulating lipid metabolism during sepsis (43).

### LPS treatment of *P. leucopus* fibroblasts.

**FIG 9:**
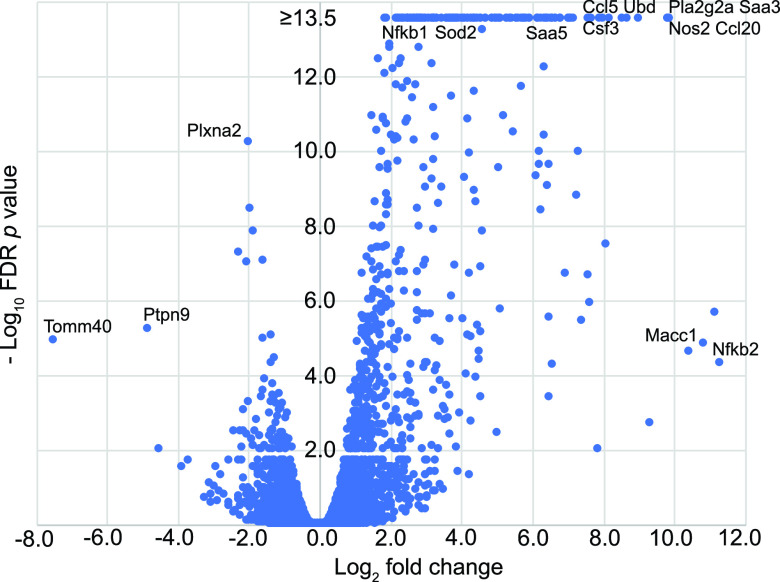
Volcano plot of RNA-seq results for pairs of *P. leucopus* fibroblast cultures with or without exposure to LPS. Fold changes for 543 genes are given on the *x* axis, and false discovery rate *P* values are given on the *y* axis. For conciseness, the upper limit for the –log_10_ values for this graph was 13.5. The exact or approximate locations of selected differentially expressed genes are shown. Numerical values for each gene in the data set are provided in the Dryad repository (https://doi.org/10.7280/D1MD69).

Low-passage-number cultures of fibroblasts, a type of stromal cell isolated from ear tissues from five LL stock deermice, were split into pairs. Then one member of each pair was treated with LPS for 4 h, and the other member was treated with saline alone. Of the 46,141 transcripts in the reference set, 18,462 had a mean TPM of >1 in either the control or the LPS group, and these were used in the DEG analysis (Dryad repository, https://doi.org/10.7280/D1MD69). For protein coding sequences, the 10 highest numbers of TPM, in order, among control samples were for ferritin heavy chain (Fth), Slpi, secreted protein acidic and cysteine rich (Sparc), eukaryotic translation elongation factor 1 alpha (Eef1a1), ferritin light chain (Ftl), vimentin (Vim), serpin family H member 1 (Serpinh1), ribosomal protein lateral stalk subunit P1 (Rplp1), collagen type I alpha 1 chain (Col1a1), and Gapdh. There were 324 genes that were upregulated by the criterion of a fold change of ≥4 and an FDR of <0.05, and 17 genes that were downregulated, with an additional 80 downregulated by the criterion of a fold change of ≥2 and an FDR of <0.5 (Fig. 9). Among those displaying the marked increases in expression between the control and LPS conditions were two subunits of nuclear factor kappa B (Nfkb), two forms of Saa, Nos2, Csf3, Sod2, and Pla2g2a, which is associated with inflammation during systemic bacterial infection (72). Slpi transcripts were not only surprisingly abundant in the fibroblast cultures under usual cultivation conditions, but expression further increased by 1.5-fold (95% CI, 1.2- to 1.9-fold) in those pair members exposed to LPS (*P = *0.02). Expression of Nos2 increased 792-fold (95% CI, 124- to 5,073-fold) in the LPS-treated fibroblasts between paired specimens (*P = *10^−5^). This was evidence that the absent or scant expression of inducible nitric oxide synthase that we observed in both control and LPS-treated *P. leucopus* animals was not attributable to a genotypic incapacity to transcribe the Nos2 gene.

### Conjunctivitis.

After identification of a variety of DEGs under conditions of LPS treatment of whole animals and isolated cells or of systemic infection, we returned to the disease sign that we observed in *P. leucopus* but not in *M. musculus*, namely, conjunctivitis severe enough to cause eyelid closure within 2 to 4 h of the LPS injection. Six of 12 animals receiving the LPS overtly manifested conjunctivitis. Table 2 lists DEGs in the blood or spleen from RNA-seq with absolute fold changes between animals with conjunctivitis and those without of >2 and FDR values of ≤0.05. Of note, in the blood of deermice with conjunctivitis, we found 21-fold-higher expression of Cxcl13, 12-fold-higher expression of Ccl6, but 46-fold-lower expression of lysozyme (with an FDR of <10^−4^), an antimicrobial enzyme and constituent of tears that coat and protect the conjunctiva (73). In spleen samples, the conjunctivitis animals was distinguished by 6- to 7-fold-higher expression (FDR, <0.005) of two forms of carbonic anhydrase, an enzyme of relevance for the eye disorder glaucoma, because of its role in producing the aqueous humor in the eye (74).

**TABLE 2:** Differentially expressed genes between 6 *P. leucopus* deermice with and 6 without conjunctivitis after LPS treatment

Specimen	Protein designation	Fold change	FDR *P* value	Product
Blood	Cxcl13	20.7	4.0E–02	C-X-C motif chemokine ligand 13
	Serpine1	17.2	4.0E–02	Serpin family E member 1
	Fn1	13.0	5.0E–02	Fibronectin 1
	Cd5l	12.9	5.0E–02	CD5 molecule like
	Ccl6	11.7	2.0E–02	C-C motif chemokine 6
	Serpinb2	9.4	4.0E–02	Serine peptidase inhibitor, clade B, member 2
	Ecm1	7.5	4.8E–03	Extracellular matrix protein 1
	Cyb5r3	5.5	1.3E–05	NADH-cytochrome b5 reductase 3
	Ecm1	5.2	2.0E–02	Extracellular matrix protein 1
	Lyz2	−45.8	5.6E–05	Lysozyme C-2

Spleen	Ahsp	14.0	4.4E–04	Alpha hemoglobin-stabilizing protein
	Slc4a1	12.5	2.0E–03	Solute carrier family 4 member 1
	Hemgn	11.6	2.0E–03	Hemogen
	Hmbs	9.0	9.5E–04	Hydroxymethylbilane synthase
	Epb42	8.8	2.7E–03	Erythrocyte membrane protein band 4.2
	Tmcc2	8.3	2.6E–03	Transmembrane and coiled-coil domain family 2
	Ca2	6.7	9.0E–04	Carbonic anhydrase 2
	Gypa	6.0	5.9E–03	Glycophorin A
	Rrm2	5.6	3.0E–04	Ribonucleotide reductase regulatory subunit M2
	Ca1	5.5	2.5E–03	Carbonic anhydrase 1
	Dhrs11	5.5	2.7E–03	Dehydrogenase/reductase 11
	Slc25a37	5.2	9.7E–03	Solute carrier family 25 member 37
	Alox15	4.9	5.0E–02	Arachidonate 15
	Fech	4.9	3.8E–03	Ferrochelatase
	Cpox	4.2	3.7E–03	Coproporphyrinogen oxidase
	Prxl2a	4.0	4.0E–02	Peroxiredoxin-like 2A
	Aqp1	3.9	1.5E–03	Aquaporin 1
	Urod	3.9	5.3E–03	Uroporphyrinogen decarboxylase
	Selenbp1	3.8	2.0E–03	Selenium binding protein 1
	Ranbp10	3.7	6.0E–03	RAN binding protein 10
	Gstm3	3.6	4.0E–03	Glutathione *S*-transferase mu 3
	Pnpo	3.5	6.9E–03	Pyridoxamine 5′-phosphate oxidase
	Xpo7	3.5	8.6E–03	Exportin 7
	Cat	3.4	6.3E–03	Catalase
	Mkrn1	3.3	5.0E–02	Makorin ring finger protein 1
	Glrx5	2.8	3.0E–02	Glutaredoxin 5
	Rad23a	2.6	1.0E–02	RAD23 homolog A, nucleotide excision repair
	Tfrc	2.4	4.0E–02	Transferrin receptor
	Hmgb2	2.4	2.0E–02	High-mobility group box 2
	Gypcc	2.2	2.0E–02	Glycophorin C
	Prdx2	2.0	4.0E–02	Peroxiredoxin 2
	Serpina3n	−2.0	7.5E–04	Serine protease inhibitor A3N
	Sirpb1b	−2.2	5.0E–02	Signal-regulatory protein beta 1
	H2–Q10	−2.3	6.2E–03	H-2 class I histocompatibility, Q10 alpha

## DISCUSSION

The study yielded abundant information about a small mammal that is emerging as an informative model organism, not only for the study of pathogenesis and immunology of infectious diseases but also for the fields of aging, behavior, ecology, and reproductive biology. The question driving this investigation was how do deermice largely avoid morbidity and mortality when infected with pathogens that otherwise are disabling if not fatal for humans? “Sickness” has various definitions, including one operationally based on behavior for animal studies (75). *P. leucopus* and *M. musculus* animals became equivalently sick by the behavioral criteria of reduced activity, mutual huddling, and lower food intake. Nevertheless, under conditions that frequently led to death or the moribund state in various strains of *M. musculus* in several studies, *P. leucopus* animals receiving the same or even much higher doses of LPS pulled through and recovered.

To address this central question, we used RNA-seq of whole blood, spleen, and liver. The study did not specifically examine differences in mRNA isoforms or small noncoding RNAs. These results were complemented by analysis of a limited set of coding sequences, specific RT-qPCR assays, direct detection of the compounds in the blood, and metabolomics. The most extensive experiment was on the responses among several young adult animals of both sexes to a single dose of LPS at approximately the LD_50_ for mice, but we obtained similar results with *P. leucopus* of considerably older age under the same conditions and with animals that had systemic bacterial infections of 5 days’ duration. While we noted some differences between the sexes of *P. leucopus* animals in the experiment, for the most part, females and males responded the same to LPS, at least in the short term and at these doses. The experiment with *P. leucopus* fibroblast cells in culture exposed to LPS showed that the some of the findings, such as high expression of Slpi, are reproduced *in vitro* and, thus, feasibly exploited by transgenic and silencing technologies.

Integration of the metabolomics results for plasma with the transcriptomics results for the three tissues was necessarily limited. The sources of some of the metabolites in the blood would likely have been organs, e.g., the adrenal glands, or gut microbiota not subjected to RNA-seq. Nevertheless, metabolomics confirmed that *P. leucopus* responded similarly to *M. musculus* in many respects to the same dose of LPS, as well as in corticosteroid biosynthesis. The evidence of greater catabolism of tryptophan by the kynurenine pathway in the LPS-treated deermice than in mice was associated with comparatively higher expression of Ido1 in the deermice.

One of the striking differences between the two species in their responses to LPS was overall predominance in the GO term and *de novo* module analyses of neutrophil-associated genes for *P. leucopus* and cytokine-associated genes for *M. musculus* (Fig. 4 and 5). The blood was the greatest contributor to the set of genes that distinguished LPS-treated *P. leucopus* animals from treated and untreated *M. musculus* animals and from control deermice, while the liver provided the majority of genes that distinguished LPS-treated *M. musculus* mice from deermice and from control mice. A limitation of the study was that complete blood cell counts were not terminally performed; this was to ensure sufficient RNA for high-coverage sequencing and plasma for untargeted metabolomics. It is possible that the prominence of neutrophil-associated GO terms in LPS-treated deermice was accounted for by higher numbers and proportions of neutrophils in the blood of deermice than in the blood of mice. But this is not likely the sole explanation. Healthy animals of the LL stock population of *P. leucopus* at the Peromyscus Genetic Stock Center (PGSC) had lower absolute numbers and proportions of neutrophils than what was reported for the BALB/c population from which our animals were drawn (76, 77) (see Materials and Methods). A study of a separate breeding colony of LL stock *P. leucopus* animals also reported comparatively low absolute numbers and proportions of neutrophils among healthy adult deermice of both sexes (78). In addition, if there was a difference between species in overall white cell activity 4 h after injection of LPS, this was not reflected in total mitochondrion reads, which represented the number of mitochondria and transcription by individual mitochondria in the blood sample. Values declined in both species from baseline by about the same proportion.

The comparatively heightened neutrophil transcriptional activity in *P. leucopus* paralleled the appearance of conjunctivitis with purulent exudates in the eyes of deermice but not mice. While increased activity of neutrophils may lead to tissue damage from elastase and other proteases and from reactive oxygen species, this may be ameliorated in the deermice by such factors as the leukocyte protease inhibitor Slpi and Sod2, a defense against oxygen radicals.

Slpi is a nonglycosylated 12-kDa cationic, cysteine-rich protein, which is known to be increased in expression in response to LPS and lipoteichoic acids and under the stimulus of TNF and IL-1β (79). It has an inhibitory effect on the formation of neutrophil extracellular traps (80). Slpi inhibits neutrophil proteases like cathepsins and elastase but also prevents degradation of the NF-κB inhibitory proteins IκBα and IκBβ through its antiprotease activity (81). Slpi knockout or deficient animals had impaired wound healing and increased inflammation (82), elevated Nos2 activity in macrophages (83), and increased susceptibility to LPS-induced shock (84).

While LPS-treated *P. leucopus* animals exhibited increased expression of the TLR signaling adapter MyD88 and downstream inflammatory cytokines, such as IL-6, TNF, and IL-1β, to approximately the same degree as in *M. musculus*, this was countered by greater expression of cytokines associated with M2 polarization, such as IL-10 and TGFβ, as well as other anti-inflammatory mediators, like annex A1 (85), in the context of lesser expression of IL-12 and IFN-γ. The GO term cytokine-mediated signaling pathway served to distinguish both LPS-treated *P. leucopus* and LPS-treated *M. musculus* animals from their untreated counterparts (Fig. 5), but the sets of constituent genes that populated the GO term for each species overlapped for only a minority (Table S4).

Another notable difference was the contrasting expression of arginase 1 and inducible nitric oxide synthase, or Nos2, in the two species. In this phenotype, which distinguishes deermice from mice under the same conditions, *P. leucopus* resembled another cricetine rodent, the golden hamster (*Mesocricetus auratus*), during infection with Leishmania donovani (86, 87). This phenotype can also be mapped to macrophage polarization, where type M1 macrophages generally feature high Nos2 and low Arg1 expression and where most varieties of type M2, or alternatively activated macrophages, are characterized by low Nos2 and high Arg1 (54). The differences between species in their IL-10/IL-12 ratios before and after LPS were also consistent with a greater disposition of *P. leucopus* to a type M2 response (Fig. 6) (66). Further categorization into the M2 subtypes recognized in mice was inconclusive at this point. Nos2 gene knockout mice are less likely to succumb to the toxicity of LPS, but at the cost of greater susceptibility to Listeria monocytogenes or to Listeria major infections (50, 88). While experimental infections with these particular intracellular pathogens have not been reported for *P. leucopus*, this species has not been notably at risk of morbidity or mortality from the obligate intracellular bacterial pathogen Anaplasma granulocytophilia (89) or the Powassan encephalitis virus (90), which utilizes *P. leucopus* as a reservoir.

Integrin-binding sialoprotein (Ibsp), also known as bone sialoprotein 2 (BSP2), is a revelatory case in this study. There were marked increases in the expression of its gene in *P. leucopus* but not in *M. musculus* upon exposure to LPS. The Ibsp protein has predominantly been associated with bone and tooth morphogenesis and not with inflammation or innate immunity. Consequently, it is not expected to be included in innate immunity and inflammation pathways or GO terms that distinguish LPS-treated from untreated animals. However, there is justification for viewing Ibsp as *P. leucopus*’ functional substitute for osteopontin or bone sialoprotein 1 (BSP1), another small integrin-binding glycoprotein that is heavily modified posttranslationally (91). Both BSP1 and BSP2 bind to alpha v beta 3 integrins, and their genes are within 100 kb of each other on chromosome 4 of humans and chromosome 22 of *P. leucopus*. Osteopontin is a biomarker of sepsis in humans (92) and reportedly acts through Stat1 degradation to inhibit Nos2 transcription (93).

Could observed differences between the two species in these experiments be attributable in part to differences in their microbiomes? The gut metagenomes were determined from preexperiment fecal pellets from the animals in this study (35). In general, the deermice and mice had similar distributions and frequencies of bacteria at the taxonomic level of the family and in the representation and proportions of different biosynthetic, metabolic, catabolic, and regulatory functions, but there were also substantive differences in the gut microbiotas between the species that plausibly account for some distinguishing responses to LPS. The first was the presence in the intestine in *P. leucopus*, but not in *M. musculus*, of a protozoan, provisionally a new species of the parabasalid genus *Tritrichomonas*. The presence of Tritrichomonas muris in some populations of the same inbred strains of *M. musculus* mice altered their cellular immune responses to other microorganisms and antigens (94). Further distinguishing the gut microbiota was the greater abundance and diversity in *P. leucopus* of *Lactobacillus* spp. (35). In two studies on *Lactobacillus* spp. as probiotics, feeding of mice with Lactobacillus paracasei or Lactobacillus plantarum resulted in less inflammation in controls when they were challenged with an influenza virus or Klebsiella pneumoniae (95, 96). In the present study, a tryptophan metabolite in the plasma that distinguished the two species was indolepropionic acid (Table S1 and Fig. S1), which in controls was ∼4-fold-greater in abundance in deermice than in mice (*P = *0.003). This compound is the metabolic product of certain intestinal bacteria (97) and reportedly has anti-inflammatory and antioxidant properties (98).

In summary, *P. leucopus* differed in many respects from *M. musculus* in these experiments in its response to LPS. Under conditions in which about half the mice were expected to die within 48 h, the deermice survived the acute insult and recovered, a phenomenon that modeled the tolerance of these animals to infection. Simple pairwise variables characterizing the two species’ different responses to LPS were the ratios of Arg1 to Nos2 transcripts, IL-10 to IL-12 transcripts, and kynurenine to tryptophan metabolites. In their outcomes after LPS exposure, the naive *P. leucopus* animals resembled *M. musculus* animals that had become accustomed to LPS by prior exposure to a low dose of LPS (99). A characteristic profile of these pretreated mice is comparatively lesser expression of proinflammatory mediators and greater expression of genes of phagocytes (100), similar to what we observed in naive *P. leucopus*.

Whether this points to a single pathway or even a single gene remains to be determined, but we doubt that the phenomenon of infection tolerance can be reduced to a simple explanation. The evidence rather is of multiple adaptive traits representing different aspects of innate immunity, metabolism, oxidative stress management, and perhaps the microbiome that serve to sustain *P. leucopus* populations amid the varied infectious agents that they face. This is also to the benefit of these agents in this trade-off, because it renders *P. leucopus* a competent vertebrate reservoir for them and one of impact for human populations in areas of endemicity.

## MATERIALS AND METHODS

### Animals.

Adult outbred *P. leucopus* of the LL stock were obtained from the Peromyscus Genetic Stock Center (PGSC) of the University of South Carolina (http://stkctr.biol.sc.edu, accessed 10 February 2021). The LL stock colony was founded with 38 animals captured near Linville, NC, between 1982 and 1985 and has been closed since 1985. Sib-matings are avoided, and complete pedigree records are kept. Animals of the LL stock have mitochondria with the same genome sequence with little or no heteroplasmy (33). Adult BALB/cAnNCrl (BALB/c) and C.B.17 strain severe combined immunodeficiency (SCID) *M. musculus* mice were purchased from Charles River. The SCID mice were used for propagation of bacteria for infections (see below). The *P. leucopus* deermice in the study were drawn from an LL stock population at the PGSC with a mean total number of white blood cells per microliter of blood of 8,600 and absolute numbers (percentages of total white blood cells) of neutrophils, lymphocytes, and monocytes of 510 (5.9%), 8,000 (93%), and 90 (1.0%), respectively, per μl (76). The BALB/c mice were obtained from a population with a reported mean of 9,250 white blood cells per μl of blood and corresponding values for neutrophils, lymphocytes, and monocytes of 1,880 (20%), 6,600 (71%), and 640 (7%), respectively, for 8- to 10-week-old animals (77). All animals in the LPS experiments spent at least 2 weeks at the UCI facilities before the experiment.

Animals were maintained in the AAALAC-accredited UC Irvine vivarium, with 2 to 5 animals per cage according to sex and on a 12-h-light/1-h-darkness lighting schedule, at a temperature of 21 to 23°C and a humidity of 30 to 70%, with water *ad libitum*, and with a diet of 8604 Teklad Rodent (Harlan Laboratories). Prior to injections, animals were lightly anesthetized with 2.5% isoflurane in the presence of 2 liters/min oxygen. The rodents were euthanized by carbon dioxide overdose and intracardiac exsanguination at the termination of the experiment or if they were moribund, unable to eat or drink, or otherwise distressed. Dissection was carried out immediately. Instruments were cleaned first and then sterilized between dissections.

The study was carried out in accordance with the recommendations in the *Guide for the Care and Use of Laboratory Animals* of the National Institutes of Health (101). University of California Irvine protocol AUP-18-020 was approved by the Institutional Animal Care and Use Committee (IACUC). The protocol for the comparative study of *P. leucopus* and *M. musculus* animals for responses to LPS after 4 h was in addition approved by the Animal Care and Use Review Office of the U.S. Army Medical Research and Materiel Command. *P. leucopus* animals studied at the PGSC were under IACUC-approved protocol 2349-101211-041917 of the University of South Carolina.

Tables A1 and A2 of the Appendix, respectively, provide information on each of the animals in the LPS experiments and corresponding National Center for Biotechnology Information (NCBI; http://ncbi.nlm.nih.gov) BioProject and BioSample identifying numbers and descriptions for these samples. The gut metagenomes from feces collected from the 20 *P. leucopus* and 20 *M. musculus* animals 1 to 2 days before the comparative experiment have previously been described (35) and are available from the MG-RAST database (https://www.mg-rast.org) under accession numbers mgm4832531 to mgm4832578.

### LPS susceptibility and dose responses.

Thirty adult *P. leucopus* deermice, divided into five groups of three females and three males, were each injected intraperitoneally (i.p.) on day 0 with a 50-μl volume of Escherichia coli O111:B4 LPS purified by ion exchange and with <1% protein and <1% RNA (Sigma-Aldrich; catalog L3024), which was diluted in sterile, endotoxin-free 0.9% saline (Sigma-Aldrich) to achieve the following doses in milligrams per kilogram of body weight: 10, 50, 100, 200, and 300. The doses were administered in randomized order over the period from 1400 to 1700 h of a single day. Animals were returned to their cages with *ad libitum* food and water and then monitored every 12 h for the following signs: reduced activity by criterion of huddling with little or no movement for >5 min, ruffled fur (piloerection), hyperpnea or rapid respiration rate, and conjunctivitis by the criterion of closed eyes with crusting observable on the eyelids. The primary endpoint was death during the period between observations or the moribund state (immobility, rapid respiration, and inability to feed or drink) at the scheduled monitoring time or when notified in the interim by vivarium attendants.

### Single-dose LPS comparison.

Animals were anesthetized with isoflurane and injected i.p. with a single dose of E. coli O111:B4 LPS at a concentration of 10 mg/kg body weight in a 50-μl volume as described above. The control group was anesthetized and then injected with the 0.9% saline alone. The experiment started at 0800 h, with 10-min intervals between animals and with alteration of LPS and control injections. At 4.0 h after their injection, the animals were euthanized. After the chest was opened, exsanguination was performed by cardiac puncture and blood was transferred to a heparin sulfate-coated tube (Becton, Dickinson Microtainer). Anticoagulated blood was centrifuged to pellet blood cells for 3 min at 4,600 × *g* at 4°C. Plasma and blood pellets were kept separately at −80°C until further analysis. Liver and spleen were extracted, flash-frozen in liquid nitrogen, and stored at −80°C until RNA extraction.

### Experimental infection.

Infection of a group of adult *P. leucopus* deermice of the LL stock with the relapsing fever agent Borrelia hermsii strain MTW of genomic group II was described by Barbour et al. (33). *Peromyscus* species are natural hosts of genomic group II strains of B. hermsii (68). In brief, animals were anesthetized and then injected on day 0 with 10^3^ bacteria divided between the i.p. and subcutaneous routes in 50-μl volumes of PBS and diluted plasma from infected SCID mice, as described previously (102). On day 4, a drop of tail vein blood was mixed with PBS and examined as a wet mount by phase-contrast microscopy to confirm infection. On day 5, animals were euthanized with carbon dioxide and terminal exsanguination. Whole blood was dispensed into heparin-coated tubes, and the spleens were removed by dissection, weighed, and then flash frozen in liquid nitrogen. Confirmation of infection and quantitation of bacterial burdens in the spleens were carried by quantitative PCR, as described previously (103).

### Fibroblast culture and LPS treatment.

Fresh ear punches were collected from five LL stock *P. leucopus* animals (2 females and 3 males) during routine marking procedures at the time of weaning at ∼3 weeks of age. The PGSC identification numbers (and mating pair for each animal) were 22608 (H-1075), 22609 (H-1075), 22610 (H-1121), 22611 (H-1121), and 22614 (H-1127). After the ear punch tissue was bathed in 70% ethanol for 2 min, it was placed in RPMI 1640 medium (HyClone FetalClone II; Thermo Scientific) supplemented with 10% fetal bovine serum (Gibco), 100 U/ml penicillin, 100 μg/ml streptomycin, and 0.292 μg/ml l-glutamine (HyClone) as previously described (104). Ear punches were minced and then treated with 2 mg/ml collagenase type I (Millipore) for 1 h. Undigested debris was removed once cells were visible. The disassociated cells were cultivated in the same medium at 37°C and in 5% CO_2_. Cells were passed when adherent layers reached 90% confluence and for no more than 7 passages. For the experiment, individual cultures were split into pairs, and they were incubated at initial concentrations of 3 × 10^5^ cells per well for 24 h. E. coli O111:B4 LPS or saline alone was added to the medium for a final LPS concentration of 1 μg/ml, and then the incubation was continued for 4 h. After disassociation of the fibroblast layer with trypsin and then addition of RNAlater (Thermo Scientific), the cells were harvested and stored at a concentration of ∼10^6^/ml in −80°C until RNA extraction.

### Analyses of blood.

Nitric oxide was analyzed from frozen plasma samples using a nitric oxide assay kit (Invitrogen) according to the manufacturer’s instructions; the nitrite concentration was calculated based on standard curve measuring optical density at 540 nm on a plate reader (BioTek Synergy2). Corticosterone was measured colorimetrically in plasma using DetectX corticosterone enzyme immunoassay kit K014-H1 (Arbor Assays). After incubation, the reaction was read at 450 nm on a BioTek Synergy2 plate reader.

### Metabolomics.

Untargeted detection and analysis of metabolites in plasma of *P. leucopus* and *M. musculus* were carried out essentially as described previously (105). In brief, 40-μl volumes of plasma, which had been stored frozen at −80°C, were extracted with 120 μl methanol containing as internal standards phenylalanine d5 (175 ng/ml), 1-methyl tryptophan (37.5 ng/ml), and arachidonoyl amide (30 ng/ml). After precipitated proteins were removed by centrifugation, the supernatant was dried under vacuum and then suspended in 50% methanol. Aliquots of 10 μl were subjected to high-pressure liquid chromatography (HPLC) and quadrupole time-of-flight mass spectrometry (Q-TOF-MS) with periodic inclusion of pooled samples for quality control. Metabolites were separated on an Agilent Technologies Poroshell C_8_ column (100 by 2.1 mm, 2.7 μm) with a gradient of acetonitrile and water, both containing 0.1% formic acid, with an Agilent 1260 HPLC pump under conditions previously described (105). The eluent was introduced into an Agilent Technologies 6520 Q-TOF-MS instrument equipped with an electrospray ionization source. The parameters for the analysis were a capillary voltage of 4,000 V, a fragmentor voltage of 120 V, gas at 310°C, a gas flow of 10 liters/min, and a nebulizer pressure of 45 lb/in^2^ (gauge). Data were acquired in the positive ion mode at a scan range of 75 to 1,700 for the mass-to-charge ratio (*m/z*) and at a rate of 1.67 spectra per second. The raw data were deposited at the Metabolomics Workbench repository (https://www.metabolomicsworkbench.org) (https://doi.org/10.21228/M8PH55).

The raw data files were converted to the XML-based mzML format with ProteoWizard (106). The files were then processed for peak picking, grouping, and retention time correction with the XCMS suite of software (107). The centWave algorithm was applied to detect chromatographic peaks with the following parameter settings: ≤30 ppm for *m/z* deviation in consecutive peaks, a signal-to-noise ratio of 10, a prefilter setting of 3 scans with peak intensities of ≥750, and 10 to 45 s for peak width. Molecular features (MF), defined by *m/z* and retention time, were grouped across samples using a bandwidth of 15 and an overlapping *m/z* slice of 0.02. Retention time correction was performed with the retcor algorithm of XCMS. Finally, peak area was normalized by the median fold normalization method as previously described (108).

Pathway enrichment analysis was performed with Mummichog network analysis software v. 2 (109), which predicts functional activity from spectral feature tables without *a priori* identification of metabolites. This was implemented online at MetaboAnalyst (https://www.metaboanalyst.ca/). The parameter settings were a mass accuracy of 20 ppm, a positive ion mode, and a cutoff *P* value of 0.05 for the Fisher’s exact test of observed and expected hits of a given pathway. Pathway enrichment in Kyoto Encyclopedia of Genes and Genomes (KEGG; https://www.genome.jp/kegg/) terms was determined separately for the *P. leucopus* and *M. musculus* sets of LPS-treated and control animals. A cross-species comparison of enrichments for pathways in common was plotted. Identifiable metabolites constituting the pathways that distinguished between LPS-treated and control animals were listed for each species. As a measure of abundance, the peak areas of selected metabolites were extracted from raw data set using Skyline software (110). Molecular features data for the 40 individual plasma samples was deposited within the Dryad data repository (https://doi.org/10.7280/D12M4N).

### RNA extractions.

Cells of freshly obtained heparinized blood were pelleted by centrifugation for 3 min at 7,000 × *g*. The plasma supernatant and cell pellet were rapidly frozen on dry ice and stored at −80°C. For extraction of RNA from blood cell pellets in LPS experiments, we used a NucleoSpin RNA blood minikit (Macherey-Nagel). Lysis buffer and proteinase K were added to the frozen pellet and shaken while the pellet thawed. RNA from liver and spleen was extracted from 30 mg of frozen tissues, mechanically homogenized, and further lysed in Buffer RLT (Qiagen) with 2-mercaptoethanol in a TissueLyser (Qiagen) instrument with 3-mm stainless steel beads. Extraction was carried out using an RNeasy minikit (Qiagen). Extraction of total RNA from blood of *P. leucopus* animals infected with B. hermsii was as described by Long et al. (34). Frozen suspensions of dissociated, cultured fibroblasts in RNAlater (ThermoFisher Scientific) were extracted with an RNeasy minikit (Qiagen). Nucleic acid concentrations were determined with a Qubit fluorometer (ThermoFisher Scientific). The quality of the extracted RNA assessed with an Agilent 2100 Bioanalyzer with the Nano RNA chip. The RNA was stored in RNase-free distilled water at −80°C.

### RT-qPCR.

Reverse transcriptase quantitative PCR (RT-qPCR) assays were developed and implemented for measurement of transcripts of genes for the following proteins of *P. leucopus*: nitric oxide synthase 2 (Nos2), arginase 1 (Arg1), secretory leukocyte peptidase inhibitor (Slpi), and glyceraldehyde 3-phosphate dehydrogenase (Gapdh). cDNA was synthesized from extracted RNA using an iScript reverse transcription kit and iScript Supermix (Bio-Rad) for qPCR, according to the manufacturer’s instructions. The reaction mix was incubated in a thermal cycler for 5 min at 25°C, 20 min at 46°C, and 1 min at 95°C. PCR of cDNA was carried out with qPCR PowerUp SYBR green master mix (Applied Biosystems) and performed in 96-well plates in a StepOnePlus real-time PCR system (Applied Biosystems) instrument. The forward and reverse primers, synthesized by Integrated DNA Technologies (San Diego, CA), were, respectively, the following: for Arg1, 5′-TCCGCTGACAACCAACTCTG and 5′-GACAGGTGTGCCAGTAGATG; for Nos2, 5′-GACTGGATTTGGCTGGTCCC and 5′-GAACACCACTTTCACCAAGAC; for Slpi, 5′-TCCCATCAGCAGACCAGTG and 5′-TTGGGAGGATTCAGCATCATACA; and for Gapdh, 5′-TCACCACCATGGAGAAGGC and 5′-GCTAAGCAGTTGGTGGTGCA. The product sizes were 352, 192, 81, and 169 bp, respectively. For all assays, the initial step was 95°C for 10 min, followed by 40 cycles. The cycle conditions for Arg1 and Nos2 were 95°C for 15 s, 52°C for 30 s, and 72°C for 60 s. For Slpi and Gapdh, they were 95°C for 15 s and 52°C for 30 s. Standards were the corresponding PCR products cloned into the E. coli plasmid vector pUC57 and stored frozen at −80°C in single-use aliquots after plasmid purification, as described above.

### RNA-seq.

Library preparation with the Illumina TruSeq mRNA stranded kit was carried out as described previously (34). The libraries were normalized and then multiplexed to achieve 12 samples per flow cell on an Illumina HiSeq 4000 instrument and 100 or 150 cycles of paired-end read chemistry at the UC Irvine Genomic High Throughput Facility. The quality of sequencing reads was analyzed using FastQC (Babraham Bioinformatics). The reads were trimmed of low-quality reads (Phred score of <15) and adapter sequence, and corrected for poor-quality bases using Trimmomatic (111). After these steps, there were overall for all the studies between 45 and 100 million reads for each of the samples. For the comparative study of 40 animals, the mean and median numbers, respectively, of reads (× 10^6^) for the three tissues were 62.9 and 62.9 for blood, 65.2 and 63.8 for spleen, and 60.5 and 59.4 for liver. Blood and liver RNA-seq outputs were 100 paired-end reads (PE100), and spleen and fibroblast RNA-seq outputs were PE150. Paired-end reads in fastq files were quantified using kallisto v. 0.46.1 (112) with the GENCODE annotation (v. 21; https://www.gencodegenes.org) for mouse and GCF_004664715.1_Pero_0.1 _rna from the NCBI for *P. leucopus* (34). The mean (95% confidence interval [95% CI]) coefficient of determination (*R^2^*) between paired replicates of 4 RNA extracts (blood of 4 LPS-treated *P. leucopus* deermice), but with independent cDNA libraries and sequencing lanes, was 0.994 (0.990 to 0.997) for 8,055 transcripts, with mean numbers of transcripts per million (TPM) being >1, while the mean *R^2^* for the 24 discordant pairs for these samples was 0.975 (0.973 to 0.977). Sequencing reads were deposited with the Sequence Read Archive (Table 3).

**TABLE 3:** BioProject, BioSample, and Sequence Read Archive accession numbers for RNA-seq

Subject	BioProject accession no.	BioSample accession no.	Sequence read archive accession no.
Blood, spleens, and livers of 2- to 3-mo-old Peromyscus leucopus LL stock deermice after injection of lipopolysaccharide (LPS) or buffer alone (control)	PRJNA643534	SAMN15445639–SAMN15445698 (20 animals × 3 tissues = 60 samples)	SRR12781556–SRR12781615
Blood, spleens, and livers of 2- to 3-mo-old Mus musculus BALB/c mice after treatment with LPS or buffer alone	PRJNA643535	SAMN15445905–SAMN15445964 (20 animals × 3 tissues = 60 samples)	SRR12782328–SRR12782387
Blood and/or spleens of 1 to 2-yr-old *P. leucopus* LL stock deermice after injection of LPS or buffer alone	PRJNA644403	SAMN15469591–SAMN15469598; SAMN16439936–SAMN16439945	SRR15769470–SRR15769487
Blood of *P. leucopus* LL stock deermice infected with Borrelia hermsii strain MTW	PRJNA508222	SAMN10522571–SAMN10522573; SAMN10522575–SAMN10522578	SRR8283810–SRR8283816
Fibroblasts from the ears of 5 *P. leucopus* LL stock deermice cultivated with or without LPS	PRJNA672217	SAMN16563388–SAMN16563397	SRR13021354–SRR13021363

### Differential expression.

We used edgeR v. 3.28.1 for DEG analysis (113). Genes were called differentially expressed if their absolute fold change between conditions was >4.0 and the false-discovery rate (FDR) was <0.05. To compare fold changes across species, we merged the output tables from the DEG analyses and retained 14,685 orthologous genes that were synonymously annotated between both species, out of a total of 24,295 annotated genes for *P. leucopus* and 35,805 for *M. musculus* (Tables S4 to S9 in the supplemental material). To screen for DEGs that varied in magnitude by species, we required an absolute fold change of >5.0 (log_2_ = 2.5) in one species and <5.0 in the other. For DEGs designated “shared” between the species, the absolute fold change was >5.0 in both. Enrichment analysis was done for each one of the gene groups, separated by up- or downregulation. Enrichment of gene ontology (GO; http://geneontology.org) terms for biological processes was computed using EnrichR (https://amp.pharm.mssm.edu/Enrichr) (114) and plotted using ggplot (v. 3.3.2) of the R package (115). The GO terms were sorted by ascending *P* value.

RNA-seq of a limited set of protein coding sequences (CDS) of both species in the same DEG analysis was carried out using CLC Genomics Workbench v. 20 (Qiagen). Paired-end reads were aligned with a length fraction of 0.4, similarity fraction of 0.9, and penalties of 3 for mismatch, insertion, or deletion to the CDS of sets of corresponding to orthologous mRNAs of *P. leucopus* and *M. musculus*. Accession numbers are given in Appendix Table A2 (Dryad repository, https://doi.org/10.7280/D1R70J). Counted reads were those that were species specific. For example, for Arg1, the coding sequences for both *P. leucopus* and *M. musculus* were in the reference set, but for *P. leucopus*, only the unique reads for Arg1 for that species were tabulated. The cross-hybridization of reads, e.g., number of *M. musculus* reads mapping to *P. leucopus* Arg1, was no more than 5% of that of the homologously mapped gene, e.g., *M. musculus* Arg1, and usually <1%. Expression values were unique reads normalized for total reads across all the samples without adjustment for reference sequence length. These were log_10_ transformed.

### WGCNA.

We used weighted gene correlation network analysis (WGCNA) to identify densely interconnected genes (modules) (116). This was applied for the 6 data sets across species and tissues by building a matrix of gene expression with genes with numbers of TPM of >1 in one or more individual. We selected a power (β) of 13 for a soft threshold for the weighted network and specified a minimum of 100 genes per module. To merge modules with similar gene expression profiles, we carried out a dynamic tree cut with an Eigengene dissimilarity threshold of 0.2 that generated the final Eigengene profiles, where Eigengene is the first principal component of the module expression matrix (117). The inferred modules were distinguished by assigned hexadecimal color code (https://www.color-hex.com/color-names.html), e.g., darkorange2. Modules, associated GO terms, and constituent genes that are not presented in this paper have been deposited with Dryad (http://datadryad.org) under the data set name “Peromyscus_WGCNA_supplement” (https://doi.org/10.7280/D1B38G).

### Statistics.

Means are presented with 95% confidence intervals. Parametric (*t* test) and nonparametric (Mann-Whitney) tests of significance were two-tailed. Unless otherwise stated, the *t* test *P* value is given. Adjustment of *P* values for multiple testing was by the Benjamini-Hochberg method (118), as implemented in edgeR, CLC Genomics Workbench (see above), or the False Discovery Rate Online Calculator (https://tools.carbocation.com/FDR). For categorical data, an exact likelihood ratio test was performed with StatXact v. 6 (Cytel statistical software). Other methods are given in software programs or suites cited above.

### Data availability.

The National Center for Biotechnology Information BioProject, BioSample, and Sequence Read Archive (SRA) identification and accession numbers for the nucleic acid sequences obtained in this study are listed in Table 3. The raw data for the metabolomics study is at https://doi.org/10.21228/M8PH55 of the Metabolomics Workbench repository (https://www.metabolomicsworkbench.org) under project PR001060.

## APPENDIX

The species and other characteristics of animals in two LPS experiments are shown in Table A1, and GenBank accession numbers for a limited set of protein coding sequences of mRNAs are shown in Table A2.

**TABLE A1:** Line listing of animals by species and other characteristics for two lipopolysaccharide (LPS) experiments^*a*^

Species, expt	Animal	Group	Sex	Age (wks)	Mass (g)	CJ	Tissues	Microbiome α-diversity^*b*^	Cortico-sterone (pg/mL)	Nitric oxide (μmol/L nitrate)	Blood MT RNA log_10_ no. of reads
Peromyscus leucopus, expt 1	22275	Control	F	15	20	–	Bld, liv, spl	3.49	254	17.3	5.64
	22277^*c*^	Control	F	15	14	–	Bld, liv, spl	3.18	238	1.1	5.76
	22282	Control	F	14	15	–	Bld, liv, spl	3.51	21	ND	5.79
	22279	Control	F	15	15	–	Bld, liv, spl	3.50	78	3.9	5.79
	22321	Control	M	13	16	–	Bld, liv, spl	3.49	214	9.8	5.43
	22335	Control	M	12	17	–	Bld, liv, spl	3.42	84	7.8	5.89
	22341	Control	M	11	15	–	Bld, liv, spl	3.80	100	3.7	5.63
	22315	Control	M	13	20	–	Bld, liv, spl	3.83	497	ND	5.68
	22258	LPS	F	17	26	–	Bld, liv, spl	3.54	681	7.7	5.52
	22281	LPS	F	15	21	+	Bld, liv, spl	3.61	748	10.2	5.75
	22278^*c*^	LPS	F	15	17	+	Bld, liv, spl	3.31	740	11.5	5.54
	22289	LPS	F	15	15	–	Bld, liv, spl	3.62	642	ND	5.70
	22292	LPS	F	15	20	–	Bld, liv, spl	3.69	817	0.0	5.74
	22284	LPS	F	15	15	+	Bld, liv, spl	3.69	671	0.0	5.75
	22319	LPS	M	13	18	–	Bld, liv, spl	3.76	697	2.7	5.61
	22316	LPS	M	13	21	+	Bld, liv, spl	3.74	713	14.7	5.62
	22325	LPS	M	12	27	–	Bld, liv, spl	3.73	697	ND	5.65
	22332	LPS	M	12	25	–	Bld, liv, spl	3.40	658	ND	5.61
	22327	LPS	M	12	15	+	Bld, liv, spl	3.77	664	4.7	5.22
	22339	LPS	M	11	20	+	Bld, liv, spl	3.87	658	11.3	5.34

Mus musculus, expt 1	102	Control	F	10	20	–	Bld, liv, spl	3.68	ND	ND	5.53
	120	Control	F	10	20	–	Bld, liv, spl	3.52	21	7.8	5.52
	F1_01	Control	F	10	20	–	Bld, liv, spl	3.65	159	4.5	5.45
	F3_02	Control	F	10	19	–	Bld, liv, spl	3.65	109	2.7	5.59
	106	Control	M	12	24	–	Bld, liv, spl	3.67	34	20.7	5.77
	115	Control	M	12	26	–	Bld, liv, spl	3.59	137	4.7	5.63
	116	Control	M	12	29	–	Bld, liv, spl	3.57	38	3.7	5.64
	M4_04	Control	M	12	31	–	Bld, liv, spl	3.62	40	7.8	5.55
	101	LPS	F	10	19	–	Bld, liv, spl	2.98	709	52.1	5.32
	103	LPS	F	10	21	–	Bld, liv, spl	3.57	699	45.6	5.40
	104	LPS	F	10	20	–	Bld, liv, spl	3.14	662	29.3	5.43
	105	LPS	F	10	19	–	Bld, liv, spl	3.47	647	42.6	5.39
	118	LPS	F	10	18	–	Bld, liv, spl	3.63	709	18.3	5.28
	F3_03	LPS	F	10	21	–	Bld, liv, spl	3.65	687	23.5	5.39
	107	LPS	M	12	29	–	Bld, liv, spl	3.69	566	8.1	5.49
	108	LPS	M	12	25	–	Bld, liv, spl	3.59	490	30.3	5.49
	109	LPS	M	12	29	–	Bld, liv, spl	3.70	634	25.5	5.38
	M2_05	LPS	M	12	27	–	Bld, liv, spl	3.66	587	12.2	5.59
	M4_06	LPS	M	12	28	–	Bld, liv, spl	3.62	518	ND	5.45
	M4_07	LPS	M	12	30	–	Bld, liv, spl	3.62	577	2.5	5.48

Peromyscus leucopus, expt 2	20694	Control	F	83	21	–	Spl	ND	ND	ND	ND
	20713	Control	F	73	23	–	Bld	ND	ND	ND	5.88
	20733^*d*^	Control	F	79	18	–	Spl	ND	ND	ND	ND
	20807^*e*^	Control	F	69	18	–	Spl	ND	ND	ND	ND
	20949	Control	F	54	19	–	Spl	ND	ND	ND	ND
	20495	Control	M	93	22	–	Bld, spl	ND	ND	ND	5.71
	20613	Control	M	84	22	–	Bld	ND	ND	ND	6.15
	20688	LPS	F	83	18	+	Spl	ND	ND	ND	ND
	20695	LPS	F	83	25	–	Spl	ND	ND	ND	ND
	20696	LPS	F	74	18	–	Bld	ND	ND	ND	5.91
	20702^*e*^	LPS	F	83	17	–	Spl	ND	ND	ND	ND
	20704	LPS	F	74	19	–	Bld	ND	ND	ND	5.74
	20732^*d*^	LPS	F	71	23	–	Bld, spl	ND	ND	ND	5.78
	20819	LPS	F	69	28	+	Spl	ND	ND	ND	ND
	20483	LPS	M	94	23	–	Bld, spl	ND	ND	ND	6.20
	20501	LPS	M	93	24	–	Bld	ND	ND	ND	6.20

a CJ, presence or absence of conjunctivitis; Bld, blood; liv, liver; spl or Spl, spleen; ND, not determined; MT, mitochondrion.

b Microbiome α-diversity values from reference 35.

c Mating pair H-106, same litter.

d Mating pair H-1056, same litter.

e Mating pair H-1055, different litter.

**TABLE A2:** GenBank accession numbers for a limited set of protein coding sequences of mRNAs

Gene product designation	Product (alternate name[s])	*P. leucopus* accession no.	*M. musculus* accession no.
Acod1	Aconitate decarboxylase 1	XM_028864652.1	NM_008392.1
Alb	Albumin	XM_028887861.1	NM_009654.4
Alox5	Arachidonate 5-lipoxygenase	XM_028864037.1	NM_009662.2
Arg1	Arginase 1	XM_028877403.1	NM_007482.3
Ccl2	C-C motif ligand chemokine 2	XM_028866979.1	NM_011333.3
Cd77	CD77 antigen (HNA-2a, NB-1)	XM_028862085.2	NM_026862.3
Cd86	CD86 antigen (Ly-5)	XM_028862269.1	NM_019388.3
Crp	C-reactive protein	XM_028865632.1	NM_007768.4
Csf2	Colony-stimulating factor 2 (granulocyte-macrophage)	XM_028879989.1	NM_009969.4
Csf3	Colony-stimulating factor 3 (granulocyte)	XM_028889425.1	NM_009971.1
Cxcl11	C-X-C motif chemokine ligand chemokine 11	XM_028881923.1	NR_038116.1
Fcgr1	Fc receptor, IgG, high affinity I (CD64)	XM_037207305.1	NM_010186.5
Fcgr2b	Fc receptor, IgG, low affinity II (CD32, Ly-1)	XM_028864328.2	NM_001077189.1
Fth1	Ferritin heavy polypeptide 1	XM_028860338.1	NM_010239.2
Gapdh	Glyceraldehyde-3-phosphate dehydrogenase	XM_028892294.1	NM_008084.3
Gata3	GATA binding protein 3	XM_028891561.1	XM_030247532.1
Gdf15	Growth differentiation factor 15	XM_006989193.2^*a*^	NM_001330687.1
Hmox1	Heme oxygenase 1	XM_028854887.1	NM_010442.2
Hp	Haptoglobin	XM_028891075.1	NM_017370.2
Ibsp	Integrin binding sialoprotein (bone sialoprotein 2)	XM_028867455.1	NM_008318.3
Ido1	Indoleamine 2,3-dioxygenase 1	XM_028887820.1	NM_008324.2
Ifnb1	Interferon beta 1	XM_028859756.1	NM_010510.1
Ifng	Interferon gamma	XM_028869658.1	NM_008337.4
Il10	Interleukin 10	XM_028876480.1	NM_010548.2
Il12	Interleukin 12	XM_028867243.1	NM_001159424.2
Il13	Interleukin 13	XM_006995904.1	NM_008355.3
Il17a	Interleukin 17A	XM_028868119.1	NM_010552.3
Il1a	Interleukin 1 alpha	XM_028893405.1	NM_010554.4
Il1b	Interleukin 1 beta	XM_028893401.1	NM_008361.4
Il1r	Interleukin 1 receptor	XM_028865796.1	XM_006495713.4
Il1rn	Interleukin 1 receptor antagonist	XM_028868677.1	NM_001039701.3
Il22	Interleukin 22	XM_028869659.1	NM_016971.2
Il4	Interleukin 4	XM_028855689.1	NM_021283.2
Il5	Interleukin 5	XM_015989306.1	NM_010558.1
Il6	Interleukin 6	XM_028864183.1	NM_031168.2
Irf5	Interferon regulatory factor 5	XM_028866208.1	NM_012057.4
Itgam	Integrin alpha-M (CD11b, CD18, Ly-4)	XM_028885647.2	NM_008401.2
Lcn2	Lipocalin 2	XM_028884808.1	NM_008491.1
Mmp8	Matrix metallopeptidase 8	XM_028890975.2	NM_008611.4
Mrc1	Mannose receptor C-type 1	XM_028870508.1	NM_008625.2
MT	Complete mitochondrial genome	MG674647	NC_005089
MT_Co1	Mitochondrial cytochrome oxidase 1	MG674647	AP014886
Nfe2l2	Nuclear factor, erythroid 2 like 2	XM_028880611.1	NM_010902.4
Nfkb	Nuclear factor kappa B subunit 2	XM_028874704.1	XM_006526743.4
Nos2	Nitric oxide synthase 2	XM_028866904.1	AF065919.1
Ptx3	Pentraxin 3	XM_028888663.1	NM_008987.3
Saa3	Serum amyloid A 3	XM_028880204.1	NM_011315.3
Slpi	Secretory leukocyte peptidase inhibitor	XM_028868627.1	NM_011414.3
Sod2	Superoxide dismutase 2	XM_028866678.1	NM_013671.3
Stat1	Signal transducer and activator of transcription 1	XM_028886666.2	XM_017319463.3
Stat2	Signal transducer and activator of transcription 2	XM_028855439.1	NM_019963.2
Stat3	Signal transducer and activator of transcription 3	XM_028856799.1	NM_213659.3
Stat4	Signal transducer and activator of transcription 4	XM_028887336.1	NM_011487.5
Tgfb1	Transforming growth factor beta 1	XM_028859890.1	NM_011577.2
Tlr2	Toll-like receptor 2	XM_028894284.1	NM_011905.3
Tlr4	Toll-like receptor 4	XM_028887864.1	NM_021297.3
Tnf	Tumor necrosis factor alpha	XM_028887933.1	NM_013693.3

a The coding sequence used for Gdf15 of *Peromyscus* is that of *P. maniculatus.*
