# Supplementary figures and images for: An Infection-Tolerant Mammalian Reservoir for Several Zoonotic Agents Broadly Counters the Inflammatory Effects of Endotoxin
Source: mBio. 2021 Apr 13;12(2):e00588-21. doi: 10.1128/mBio.00588-21 (PMC8092257; doi:10.1128/mBio.00588-21)

A. Blood

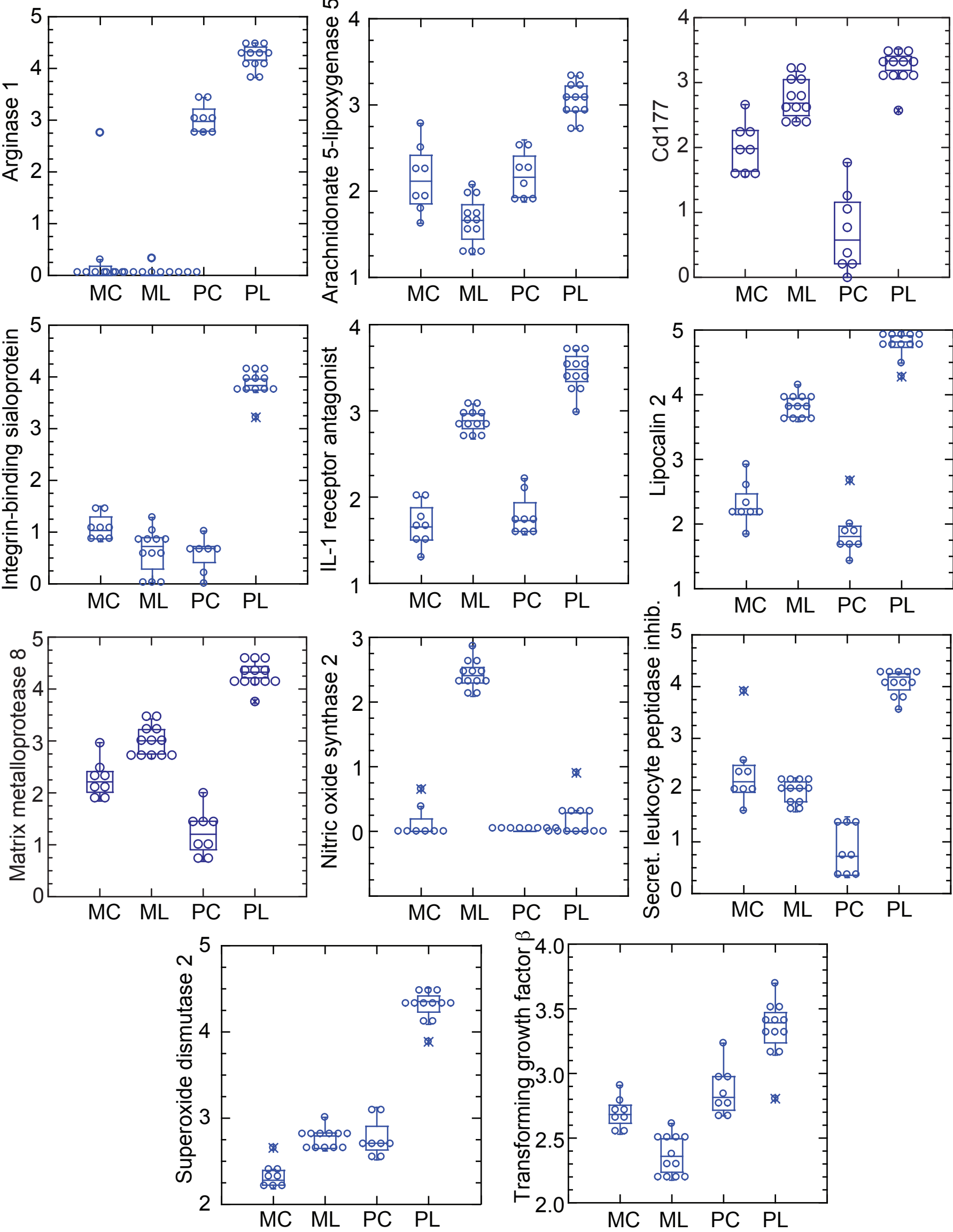

B. Spleen

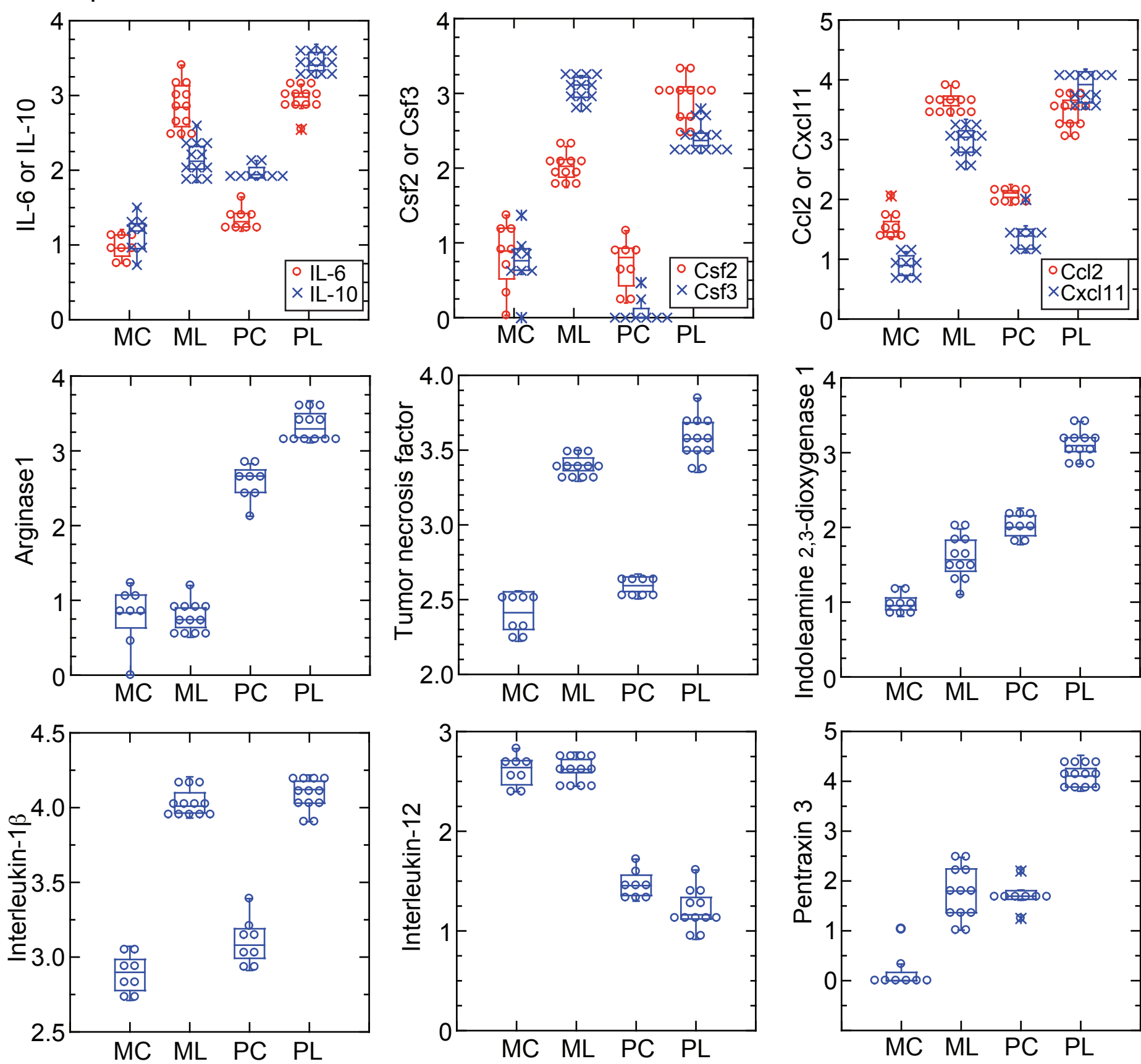

C. Liver

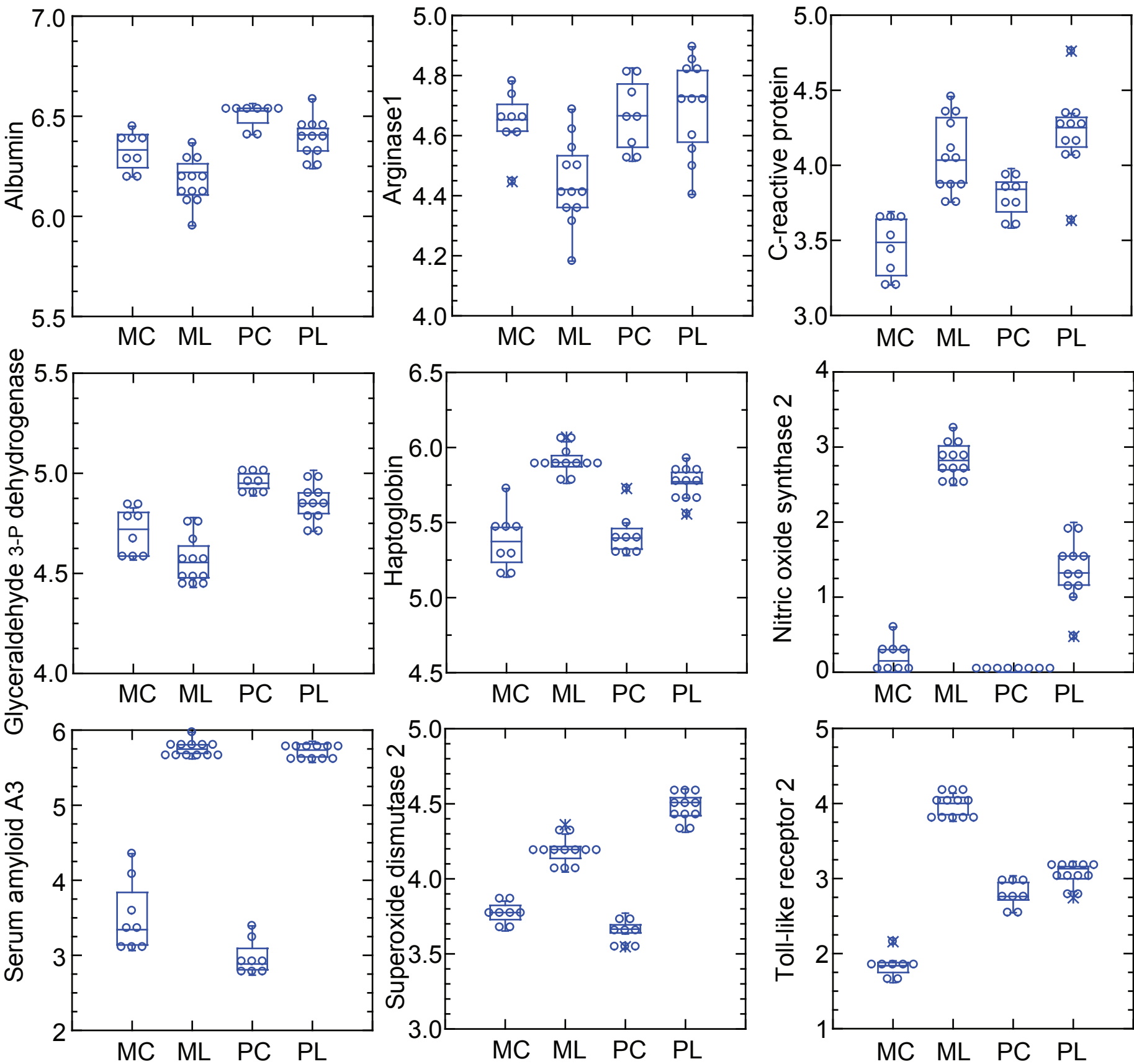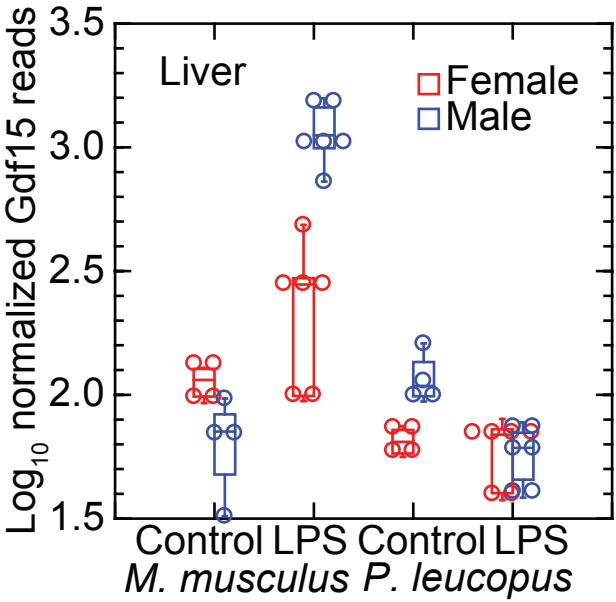

Supplement: FIG S2 [file mBio.00588-21_sf002.pdf]

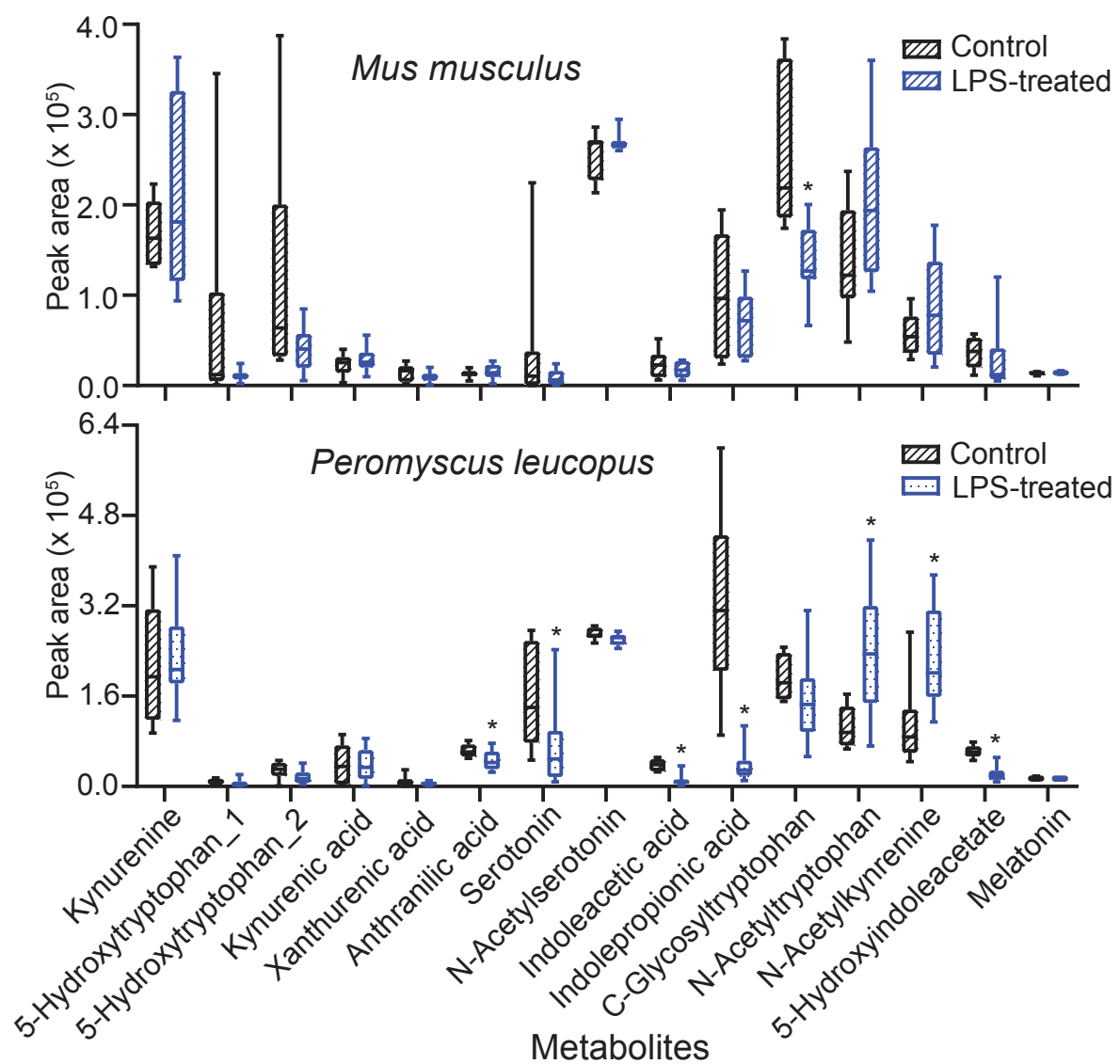

Supplement: FIG S1 [file mBio.00588-21_sf001.pdf]

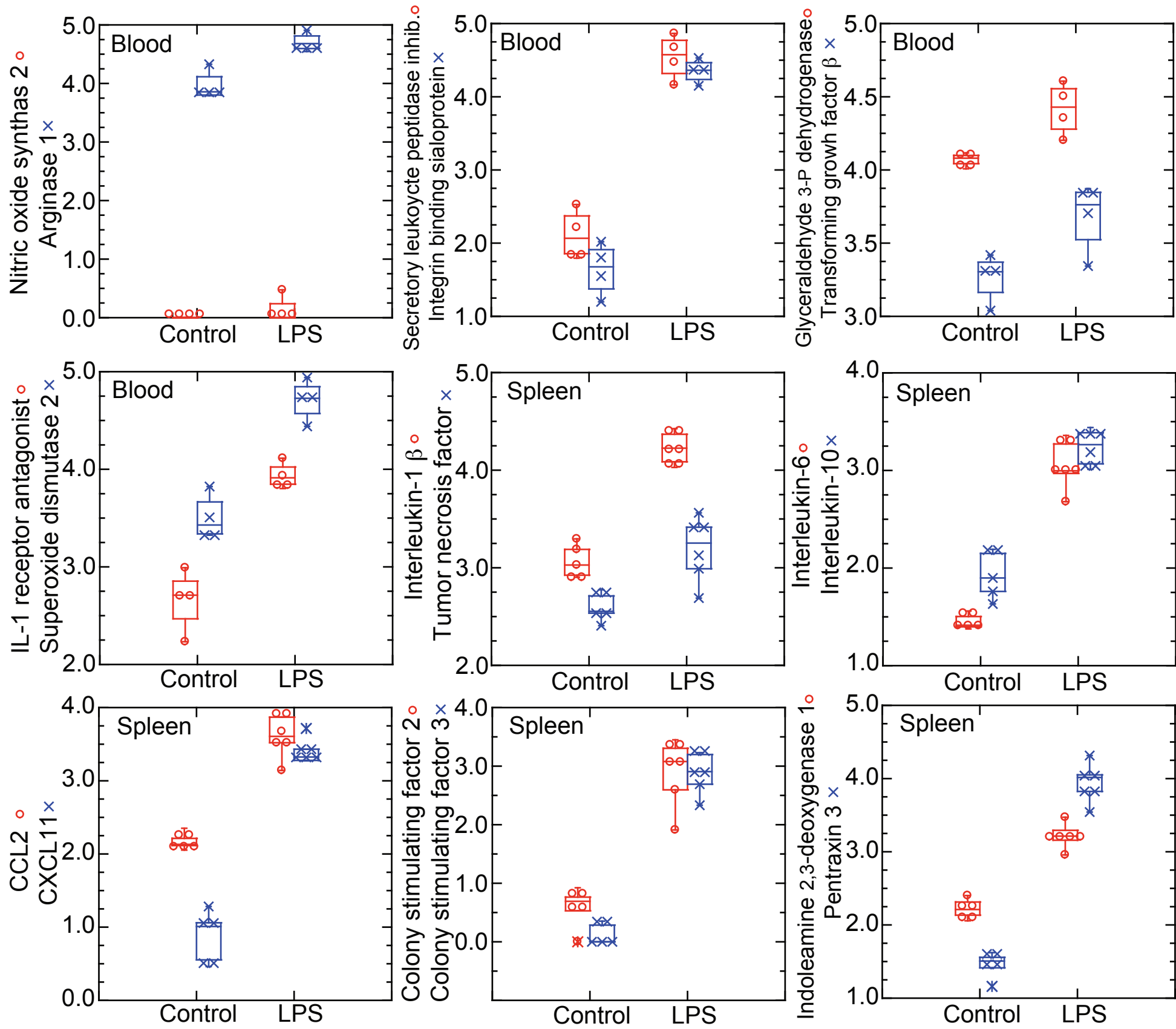

Supplement: FIG S3 [file mBio.00588-21_sf003.pdf]
